# Stochastic Analysis Demonstrates the Dual Role of Hfq in Chaperoning *E. coli* Sugar Shock Response

**DOI:** 10.3389/fmolb.2020.593826

**Published:** 2020-12-23

**Authors:** David M. Bianchi, Troy A. Brier, Anustup Poddar, Muhammad S. Azam, Carin K. Vanderpool, Taekjip Ha, Zaida Luthey-Schulten

**Affiliations:** ^1^Department of Chemistry, University of Illinois at Urbana-Champaign, Urbana, IL, United States; ^2^Center for the Physics of Living Cells, University of Illinois at Urbana-Champaign, Urbana, IL, United States; ^3^Department of Biophysics, Johns Hopkins University, Baltimore, MD, United States; ^4^HHMI Investigator Program, Howard Hughes Medical Institute, Chevy Chase, MD, United States; ^5^Department of Microbiology, University of Illinois at Urbana-Champaign, Urbana, IL, United States

**Keywords:** stochastic biology, cell simulations, small RNA, single-molecule techniques, super-resolution microscopy, gene regulatory networks, cellular stress response

## Abstract

Small RNAs (sRNAs) play a crucial role in the regulation of bacterial gene expression by silencing the translation of target mRNAs. SgrS is an sRNA that relieves glucose-phosphate stress, or “sugar shock” in *E. coli*. The power of single cell measurements is their ability to obtain population level statistics that illustrate cell-to-cell variation. Here, we utilize single molecule super-resolution microscopy in single *E. coli* cells coupled with stochastic modeling to analyze glucose-phosphate stress regulation by SgrS. We present a kinetic model that captures the combined effects of transcriptional regulation, gene replication and chaperone mediated RNA silencing in the SgrS regulatory network. This more complete kinetic description, simulated stochastically, recapitulates experimentally observed cellular heterogeneity and characterizes the binding of SgrS to the chaperone protein Hfq as a slow process that not only stabilizes SgrS but also may be critical in restructuring the sRNA to facilitate association with its target *ptsG* mRNA.

## 1. Introduction

The ability of living cells to modulate their gene expression in response to changing environmental conditions is critical to their growth and continued development. Many bacteria use the phosphoenolpyruvate phosphotransferase (PTS) system to transport and phosphorylate incoming sugars to prepare them for subsequent glycolytic metabolism. The uptake of phosphosugars must be balanced with their breakdown in order to prevent metabolic stress. In *E. coli*, a stress response induced by unbalanced glucose-phosphate transport and metabolism or “sugar shock,” is referred to as glucose-phosphate stress response. A primary activity of this stress response is RNA silencing of *ptsG*, a gene coding for the glucose transport protein of the same name (also known as EIICBGlc in *E. coli*), by the small RNA (sRNA) SgrS. Small RNAs are usually non-coding RNA molecules that act by base pairing with target messengers to regulate translation or mRNA stability and have been observed across all domains of life (Babski et al., 2014). *sgrS* is upregulated by a transcriptional activator (SgrR) when the cell is under a state of glucose-phosphate stress. SgrS regulates *ptsG* post-transcriptionally by a mechanism where SgrS binds to *ptsG* messenger RNA (mRNA) and prevents its translation to protein by blocking access of the ribosome to the mRNA (Vanderpool and Gottesman, 2004; Maki et al., 2010). This also enhances the co-degradation of *ptsG* mRNA and SgrS via enzymes responsible for the removal of bulk RNA such as ribonuclease E (RNase E) (Kawamoto et al., 2006; Maki et al., 2010). This co-degradation reduces the number of PtsG sugar transporter proteins that are produced and thus reduces the impact of glucose-phosphate stress, since fewer transport proteins are available to bring sugar into the cell.

SgrS and *ptsG* mRNA associate via complementary base pairing that occludes the ribosome binding site on the mRNA. Recently, this mechanism has been analyzed in conjunction with binding of the Sm-like chaperone protein Hfq to SgrS, which has been proposed to stabilize the sRNA, and facilitate the interaction between the sRNA and its mRNA target (Ishikawa et al., 2012). Hfq also promotes SgrS–dependent regulation of other targets involved in sugar shock such as *manXYZ*, and *yigL* in *E. coli*. In this study, we focus only on the primary regulatory target *ptsG* mRNA and do not consider the other targets of the SgrS regulon, which are described in Bobrovskyy et al. (2019).

Previous experimental and theoretical work (Jones et al., 2014; Peterson et al., 2015) has demonstrated the necessity of accounting for gene replication over the course of the cell cycle in order to capture the population variation observed in messenger RNA abundance. The additional noise emanating from transcription at multiple gene loci manifests itself in the broad mRNA copy number distributions observed in a population of cells. The aforementioned work also demonstrated that including the effect of gene regulation by transcription factors can be critical in order to appropriately describe stochastic dynamics. The effect of transcriptional regulation is apparent in the SgrS–*ptsG* mRNA system, where the expression of SgrS is maintained by the regulator SgrR, which activates *sgrS* and autorepresses its own expression during glucose-phosphate stress conditions (Vanderpool and Gottesman, 2004, 2007).

Recently, Fei et al. (2015) presented a deterministic kinetic model of the SgrS mediated regulation of *ptsG* mRNA in *E. coli*. Using single-molecule fluorescence experiments (smFISH and STORM), SgrS and *ptsG* mRNA copy numbers in cells were measured, which produced distributions of RNA at various time points after the induction of sugar stress across a population of fast-growing *E. coli*. However, it is important to note that both the *ptsG* mRNA and the SgrS regulating it are present in low copy number (a few to tens of particles) and therefore exhibit intrinsically noisy behavior in both their gene expression and regulatory behaviors. For this reason it is most appropriate to treat the regulatory processes via stochastic simulation in order to quantify the variation that is observed across a population of cells, which has been demonstrated previously (Elowitz et al., 2002; Raser, 2005; Earnest et al., 2018).

Here, we have developed a stochastic model, to our knowledge the first of its kind for an RNA silencing network, that captures the mRNA and sRNA distributions experimentally observed in a population of hundreds of *E. coli* cells. The stochastic model additionally incorporates the following features that extend the platform given by Fei et al. (2015): (1) accounting for gene replication, (2) transcriptional gene regulation of *sgrS* by its activator SgrR and (3) explicit representation of the SgrS stabilization via the Hfq chaperone protein. This model robustly describes experimentally observed RNA distributions, closely matching regulatory dynamics from immediately after induction until a steady state is reached 20 min later. We also utilize this model to analyze the effects of the size of the pool of Hfq chaperone protein available to SgrS, to decouple the rate of Hfq stabilization of SgrS and its subsequent activity in enhancing association to the target, *ptsG* mRNA, and to study the effect of an *sgrS* point mutation in the SgrS-Hfq binding region on regulatory dynamics.

## 2. Materials and Methods

### 2.1. Model and Computational Methods

The previous kinetic model for SgrS regulation of *ptsG* mRNA (Fei et al., 2015) utilized simple mass-action kinetics to describe the target search process and modeled gene expression as a constitutive process, with RNA species originating from a single gene copy. Despite its simplicity, this model captures average regulatory network behavior and also gives insight into many of the parameters required for the more descriptive stochastic model that is the focus of this work. For example, since an overall binding rate for SgrS to *ptsG* mRNA was established in Fei et al. (2015) we are now able to complexify the model by the addition of the chaperone protein Hfq, which allowed us to predict (by fitting to the experimental data) the size of the pool of Hfq available to stabilize SgrS and the rate at which it binds the sRNA (separate from its association to *ptsG* mRNA).

**Figure 1:**
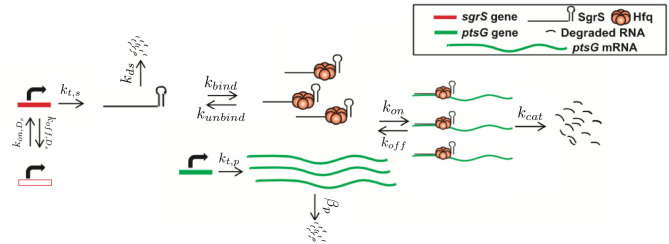
Schematic of the kinetic model as described in the text. The RNA species are transcribed from a sampled genome state with *sgrS* capable of switching between an “ON” and “OFF” state. Explicitly represented Hfq can bind and unbind with SgrS, and then the Hfq–SgrS complex binds (and potentially unbinds) with *ptsG* mRNA. All RNA degradation events are carried out by the enzyme RNase E. See Figure 4 for the kinetic equations described above.

The kinetic model was implemented and solved stochastically as a well-mixed Chemical Master Equation (CME) in the Lattice Microbes (LM) simulation software suite (Peterson et al., 2013; Roberts et al., 2013; Hallock et al., 2014; Hallock and Luthey-Schulten, 2016). The corresponding rate constants (Table 1) were adapted from the kinetic model described in Figure 1. One important feature added to the model is the explicit presence of the chaperone protein Hfq, which has been shown to both stabilize SgrS (substantially increasing its half-life) and to facilitate the association of SgrS to *ptsG* mRNA (Vanderpool and Gottesman, 2004; Hopkins et al., 2011; Wagner, 2013; Santiago-Frangos and Woodson, 2018). In order to capture the cell-to-cell heterogeneity due to the small number of particles (e.g., gene copies) involved in transcription, it is critical to account for transcriptional regulation of the genes involved in the glucose-phosphate stress response. For this reason, we include the transcriptional activation of *sgrS* by the transcription factor SgrR, which has been shown to upregulate *sgrS* expression in the presence of αMG (the unmetabolizable inducer used in place of glucose for our experiments) (Vanderpool and Gottesman, 2004, 2007). Regulation of *ptsG* by the transcriptional repressor Mlc was not included in the model since repression is relieved in the presence of glucoside sugars. With αMG present, Mlc is sequestered at the membrane by binding the EIIB subunit of the PtsG transporter protein complex (Lee, 2000; Seitz et al., 2003; Nam et al., 2008), relieving repression and resulting in high levels of *ptsG* transcriptional activity (Balasubramanian and Vanderpool, 2013). Since the decay time of PtsG proteins is expected to be approximately on the order of 8 h (Maier et al., 2011), much longer than the timescale of mRNA decay, Mlc repressors are likely still sequestered by the transporters at the membrane 20 min post-induction and have little effect on the SgrS regulatory process. Rates for the association of the Hfq-SgrS complex to *ptsG* mRNA (*k*_*on*_) and the dissociation of the Hfq-SgrS-*ptsG* mRNA complex (*k*_*off*_) were obtained from Fei et al. (2015), which did not include Hfq explicitly but provides the corresponding association and dissociation reaction rates. The value for the co-degradation rate of SgrS and *ptsG* mRNA from the Hfq-SgrS-*ptsG* mRNA complex by RNase E (*k*_*cat*_) is also obtained from Fei et al. (2015) (see section 2.2 for confirmation of *k*_*on*_, *k*_*off*_, and *k*_*cat*_ values).

**Table 1:** The list of parameters used for the kinetic model.

**Parameter**	**Value**	**Unit**	**Source**
*k*_*t, p*_	0.12 ± 0.01	*s*^−1^	Experimentally measured
β_*p*_	(3.7 ± 0.5) ×10^−3^	*s*^−1^	Experimentally measured
*k*_*on*,_*D*__*s*__	(3.0 ± 0.1) ×10^−2^	*s*^−1^	Fit
*k*_*off*,_*D*__*s*__	(9.5 ± 0.1) ×10^−3^	*s*^−1^	Fit
*k*_*t, s*_	0.33 ± 0.01	*s*^−1^	Fit
*k*_*ds*_	0.022 ± 0.002	*s*^−1^	Δ*hfq* decay rate of SgrS
*k*_*bind*_	0.063^*a*^ ± 0.014	*s*^−1^	Fit
*k*_*unbind*_	0.0018 ± 0.0004	*s*^−1^	SgrS decay rate
*k*_*on*_	(3.1± 0.2) ×10^−4^	*molec*^−1^ *s*^−1^	Fei et al., 2015
*k*_*off*_	0.22 ± 0.02	*s*^−1^	Fei et al., 2015
*k*_*cat*_	0.3 ± 0.1	*s*^−1^	Fei et al., 2015
% high, low gene state *sgrS*	25 ± 12, 75 ± 12	%	Fit
% high, low gene state *ptsG*	46 ± 20, 54 ± 20	%	Fit
Hfq pool size (available to SgrS Regulon)	250 ± 167	*molec*	Fit

The % in each gene state refers to percentage of the cellular population with the gene being in a low or high gene copy state as described in section 2.1.1. (a) k_bind_ is given as a Pseudo first order rate accounting for the average expected pool size of Hfq participating in SgrS stabilization and enhancement (250). When converted to the corresponding bulk second order rate with 250 Hfq present k_bind_ agrees well with the range of Hfq binding rates measured for other sRNA reviewed in Santiago-Frangos and Woodson (2018) and discussed further in section 3. Confirmation of k_on_ and k_off_ as the same values given in Fei et al. (2015) is discussed in section 2.2. Calculation and analysis of parameter uncertainty values by Markov Chain Monte Carlo analysis is discussed in Supplementary Section 6.

#### 2.1.1. Calculation of Gene Copy Number

**Figure 2:**
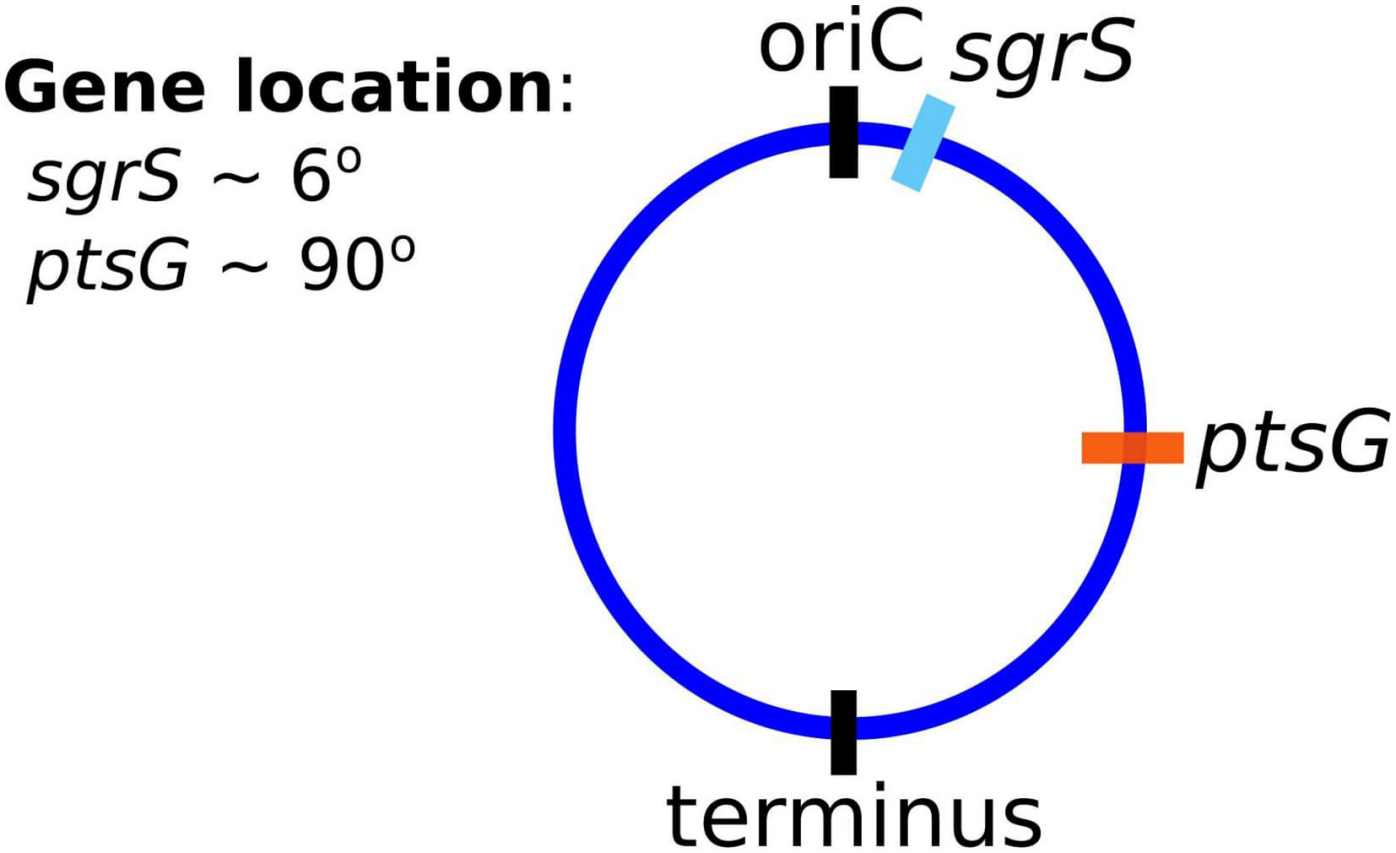
The gene location for SgrS and *ptsG* mRNA relative to the origin of replication (*oriC*) are shown on the circular genome of the *E. coli* cells used for this study. As it is closer to the origin of replication *sgrS* (cyan) is likely to be present in higher gene copy number than *ptsG* (orange), which is farther away from the *oriC*.

Finally, and critically, in order to appropriately capture regulatory effects on gene expression of SgrS and *ptsG* mRNA, it is important to account for gene duplication, as we have previously shown (Peterson et al., 2015). As illustrated by Jones et al. (2014) since the time to replicate the *E. coli* genome (approximately 40 min, Cooper and Helmstetter, 1968) is longer than the fast-growing *E. coli* cell division time of 20 min (or the 35 min observed in our experiments), the cell has nested replication forks that are already replicating the genomes of daughter and granddaughter cells prior to cell division. In particular, genes close to the origin of replication are likely to have multiple copies present over much of the cell cycle. This phenomenon has been shown previously for genes near the origin in *E. coli* by both isotopic labeling of nucleotides and imaging of fluorescent chromosome markers (Cooper and Helmstetter, 1968; Youngren et al., 2014). Due to the position of *sgrS* (only 6° away along the circular chromosome) very near to the origin of replication, it is likely that multiple gene copies are accessible for transcription over the course of the cell cycle. About half-way between the origin and terminus of replication (at approximately 90°) *ptsG* is also likely to have multiple gene copies present at some point over the course of the cell cycle, although at lower copy number than *sgrS*. Figure 2 depicts the two genes and their location along the circular *E. coli* genome.

The experimentally measured cells were unsynchronized and should have multiple replication forks present over the course of the 20 min post-induction, our measurement window. To account for gene duplication effects in a population of unsynchronized cells, we sample the percentage of the cellular population in either a low or high gene state, which corresponds to the expected distribution of the number of genes present over the course of the cell cycle after induction. In this way, we effectively flip a coin to decide whether a simulation replicate corresponding to an individual experimentally imaged *E. coli* cell has 2 copies (low gene state) or 4 copies (high gene state) of *sgrS* and similarly 1 or 2 copies of *ptsG*. This allows us to account for the effect of gene duplication in generating mRNA noise over the heterogeneous population of hundreds of *E. coli* cells that were observed experimentally. We assume that all gene copies are transcribed independently from one another and at the same rate, a notion that Wang et al. (2019) has recently examined in *E. coli* under various growth conditions. Under similar growth conditions to ours [MOPS glucose-based medium with a doubling time of 35 min (see section 2.2)], the data from Wang et al. (2019) suggest that transcription does appear to be independent and uncorrelated between copies of the same gene.

**Figure 3:**
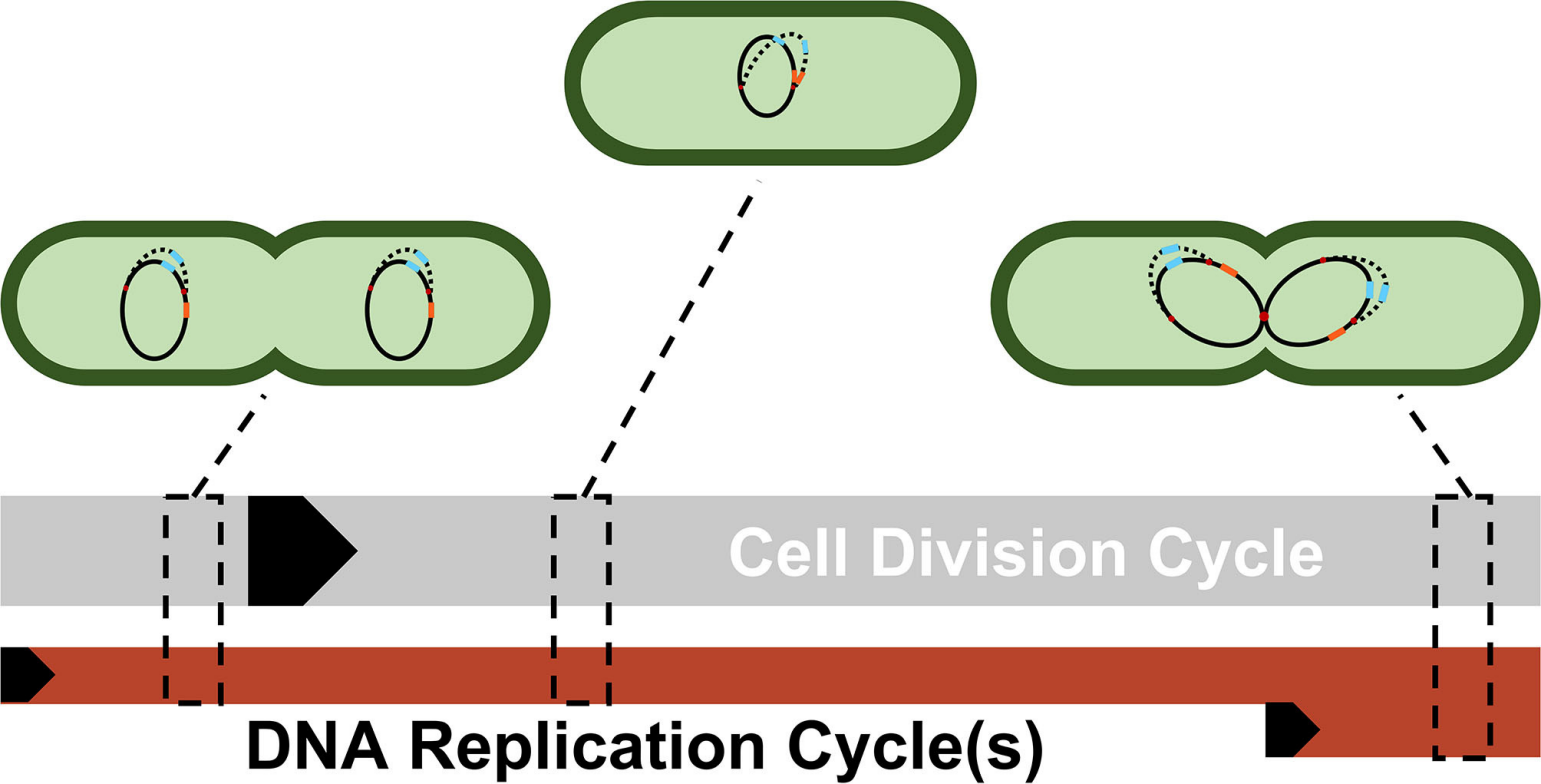
A simplified depiction of possible cellular states throughout a single DNA replication cycle. Each cell shows a snapshot of the gene state of a cell given its progression through the DNA replication and cell division cycle. Due to the difference in lengths of the cell division cycle (~35 min) and DNA replication cycle (~40 min), DNA replication and cell division are not completely in sync. Multiple replication forks (red dots) can form on the genome in order to ensure DNA is duplicated properly in these fast-growing cells. As a result, genes closer to the origin such as *sgrS* (blue) are duplicated in the same timeframe that replication is initiated (resulting in 2 or 4 gene copies), while genes closer to the terminus such as *ptsG* (orange) are replicated later in the C period, the period when a majority of DNA is duplicated (resulting in 1 or 2 gene copies). The black arrows denote the start of a cycle.

Figure 3 illustrates the reasoning for the specific choices of high and low state gene copy numbers for *ptsG* and *sgrS* in an *E. coli* cell growing faster than the expected time necessary for replication (approximately 40 min, compared to an experimentally observed generation time of approximately 35 min) (Cooper and Helmstetter, 1968; Youngren et al., 2014).

Stochastic simulations were performed by sampling the CME for the model given in Figure 1 with the widely used Gillespie Direct Method of the Stochastic Simulation Algorithm (SSA), which is implemented in the publicly available Lattice Microbes (LM) software suite (version 2.3 was used) and its python interface pyLM (Peterson et al., 2013; Roberts et al., 2013; Hallock et al., 2014; Hallock and Luthey-Schulten, 2016). We ran 2,000 replicate simulations for 25 min after αMG induction of glucose-phosphate stress in order to match the corresponding 20 min smFISH-STORM experiments. Initial conditions for basal SgrS (1–3 copies) and *ptsG* mRNA (30–40 copies) copy number were sampled from the experimentally measured distributions and rounded to the nearest integer particle number (a necessity for stochastic representation). Simulations were computed on a local cluster containing AMD Opteron Interlagos cores.

#### 2.1.2. SgrS Regulatory Network Kinetic Model

**Figure 4:**
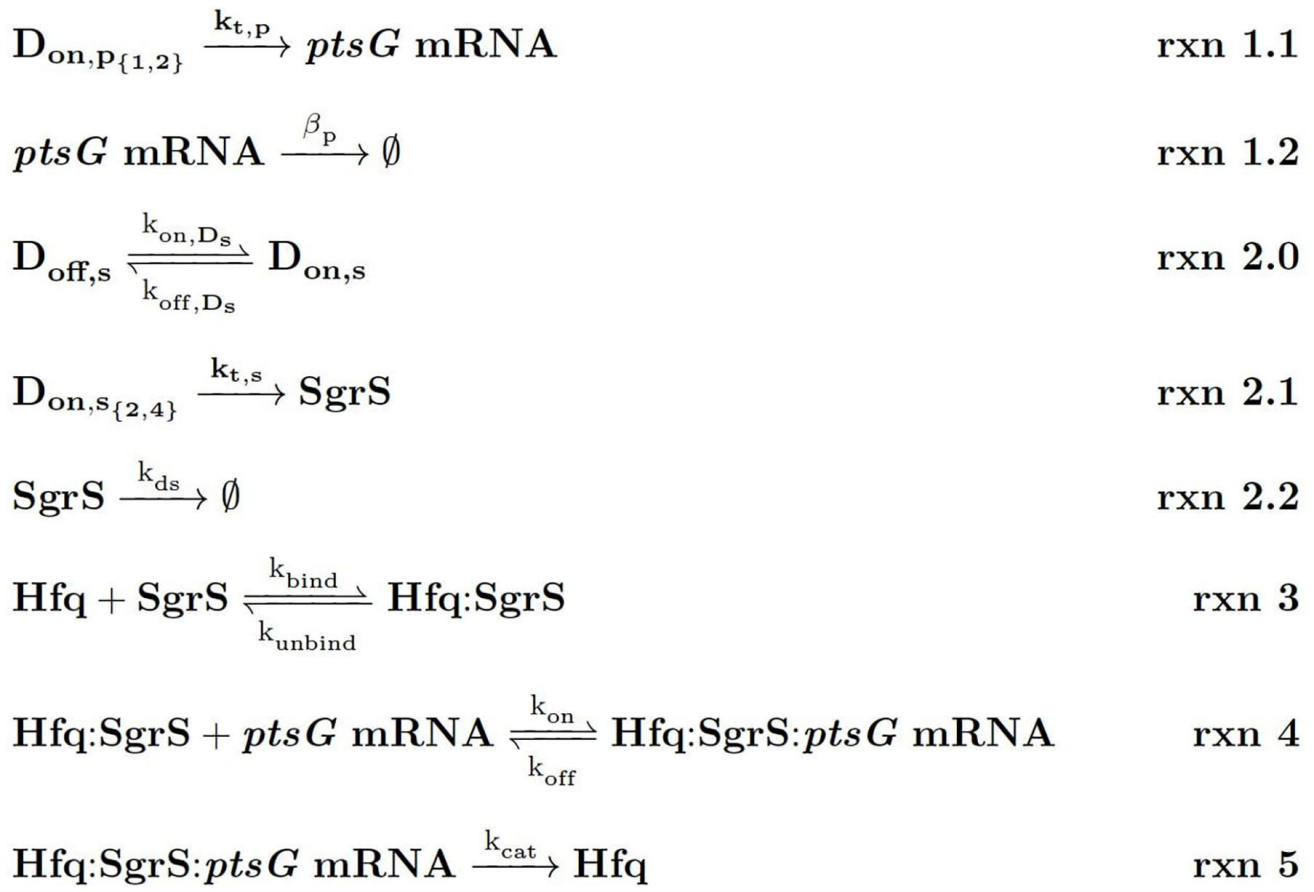
Kinetic Equations of the SgrS regulatory network. *D*_*on*,_*p*__1,2__ refers to the gene (or DNA) for *ptsG* in 1 (low state) or 2 (high state) copies and *D*_*on*,_*s*__2,4__ corresponds to the gene for *sgrS* in 2 (low state) or 4 (high state) copies. *D*_*on,s*_ corresponds to *sgrS* when it is in the “ON” state due to activated or solute bound transcriptional activator SgrR being bound (Vanderpool and Gottesman, 2007). *k*_*ds*_ corresponds to the experimentally measured degradation rate of SgrS when cellular Hfq is not present and *k*_*unbind*_ corresponds to the experimentally measured degradation of SgrS when Hfq was present.

The kinetic model describing the reactions characterizing the *E. coli* glucose-phosphate response network by the small RNA SgrS is given in Figure 4. Simulation files are available in Jupyter Notebook format to be simulated via the Lattice Microbes (LM) Software Package at http://faculty.scs.illinois.edu/schulten/research/sgrs-2020/.

### 2.2. Experimental Methods and Materials

Wild type *E. coli* cells (DJ480) were grown overnight at 37 °C, 250 rpm in LB Broth. The SgrS U224G mutant was grown in LB Broth with 50 μg/ml spectinomycin (Spec) (Sigma-Aldrich). The next day, overnight cultures were diluted 100-fold into MOPS EZ rich defined medium with 0.2% glucose and the cells were grown until *OD*_600_ reached 0.15–0.25. α-methyl D-glucopyranoside (αMG) (Sigma Aldrich) was then added to provoke glucose-phosphate stress and induce SgrS expression response. Specific volumes of liquid were removed from the culture at 0, 2, 4, 6, 8, 10, 15, and 20 min after induction and mixed with formaldehyde (Fisher Scientific) to a final concentration of 4% for cell fixation prior to single molecule experiments. See Supplementary Table 1 for a description of the cellular strains utilized for these experiments.

Following fixation, the cells were incubated and washed, before being permeabilized with 70% ethanol, to allow for fluorescence *in situ* hybridization (FISH). Stellaris Probe Designer was used to design the smFISH oligonucleotide probes that were ordered from Biosearch Technologies (https://www.biosearchtech.com/). See Supplementary Table 2 for a table of the probes used in this work. Each sRNA was labeled with 9 Alexa Fluor 647 probes while each *ptsG* mRNA was labeled with 28 CF 568 probes. The labeled RNA molecules were then imaged via the super-resolution technique STORM (Stochastic Optical Reconstruction Microscopy). A density-based clustering analysis algorithm (DBSCAN) (Daszykowski et al., 2001) was utilized to calculate RNA copy numbers. The algorithm used was the same as previously published (Fei et al., 2015), but the Nps and Eps values were updated for the SgrS and *ptsG* mRNA images, since CF 568 was used instead of Alexa Fluor 568 and a 405 nm laser was used to reactivate the dyes. The SgrS (9 probes labeled with AlexaFluor 647) images were clustered using *Nps* = 3 and *Eps* = 15 and the *ptsG* mRNA (28 probes labeled with CF 568) images were clustered using *Nps* = 10 and *Eps* = 25 and these numbers were empirically chosen. A MATLAB code was used for cluster analysis.

The raw data was acquired using the Python-based acquisition software and it was analyzed using a data analysis algorithm which was based on work previously published by Babcock et al. (2013). The peak identification and fitting were performed using the method described previously (Fei et al., 2015). The z-stabilization was done by the CRISP system and the horizontal drift was calculated using Fast Fourier Transformation (FFT) on the reconstructed images of subsets of the super-resolution image, comparing the center of the transformed images and corrected using linear interpolation.

The *ptsG* mRNA degradation rates were calculated via a rifampicin-chase experiment. The wild type (DJ480) *E. coli* cells and Δ*hfq* mutant strain SA1816 [DJ480, *laclg, tetR, spec*, Δ*hfq::kan*] cells were grown in LB Broth with the respective antibiotics at 37 °C, 250 rpm overnight. They were used to calculate the RNA degradation rates. The Δ*hfq::kan* allele was moved to create strain SA1816 constructed by P1 transduction (Miller, 1972). When the *OD*_600_ reached 0.15–0.25, rifampicin (Sigma-Aldrich) was added to cultures to a final concentration of 500 μg/ml. The cells were labeled by smFISH probes and analyzed by the same process described above, taking the time of rifampicin addition or αMG removal as the 0 time point. Aliquots were taken after 0, 2, 4, 6, 8, 10, 15, and 20 min (0, 2, 4, 6, and 8 min for ΔHfq strains). For the purpose of background subtraction, ΔSgrS and Δ*ptsG* mRNA strains were grown, labeled with probes and imaged in the same manner to be used for the measurement of the background signal due to the non-specific binding of Alexa Fluor 647 and CF 568. The natural logs of the RNA copy numbers were plotted against time and the slope of the linear fitting was used to calculate the RNA lifetime and then the degradation rates. SgrS degradation rates were obtained from Fei et al. (2015), where they were measured by stopping the transcription of *sgrS* by removing αMG from the media and then were imaged and analyzed to calculate the degradation rates in the same manner as was described for *ptsG* mRNA. The values for *k*_*cat*_, *k*_*on*_, and *k*_*off*_ for WT cells were confirmed to be within the errors reported for the values given in (Fei et al., 2015) by fitting to the experimentally measured RNA counts with the simplified model given in that work. The transcription rate of *ptsG* was determined using *k*_*t*.*p*_ = β_*p*_ × [*p*]_0_, [as described in Fei et al. (2015)], where [*p*]_0_ was the average initial level of *ptsG* mRNA before stress induction. The transcription rate obtained was unchanged between the wild-type and the U224G mutant cells.

## 3. Results

**Figure 5:**
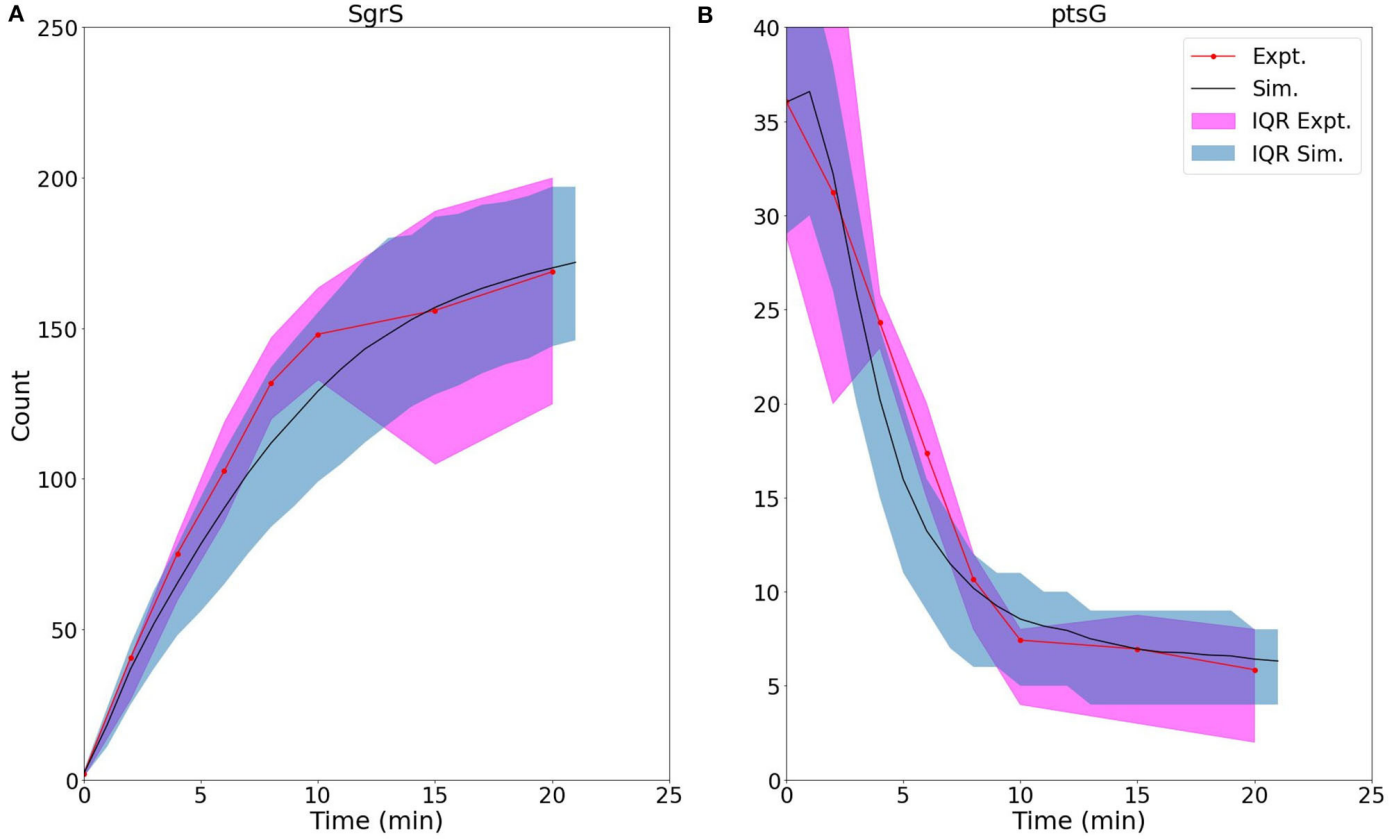
Average time trace and interquartile range (IQR) of **(A)** labeled SgrS and **(B)** *ptsG* mRNA from both 85–169 cells from smFISH experiments (red, circled line) and 2,000 replicates from kinetic model simulations (blue, solid line). The kinetic model shows strong agreement, especially at long times (10–20 min) after induction and captures overall response behavior. An available pool of 250 Hfq and the kinetic parameters given in Table 1 were utilized. Results considering both lower and higher available Hfq pools are discussed in Supplementary Figure 1.

Figure 5 demonstrates the ability of our newly developed kinetic model to capture the average cellular copy number of SgrS and *ptsG* mRNA over the course of the 20 min period post-induction. The overlap of the interquartile range (IQR) of both the experimental and simulated cellular populations demonstrates the agreement over a variety of cells [at different gene states (i.e., high/low copy number), and RNA expression levels].

**Figure 6:**
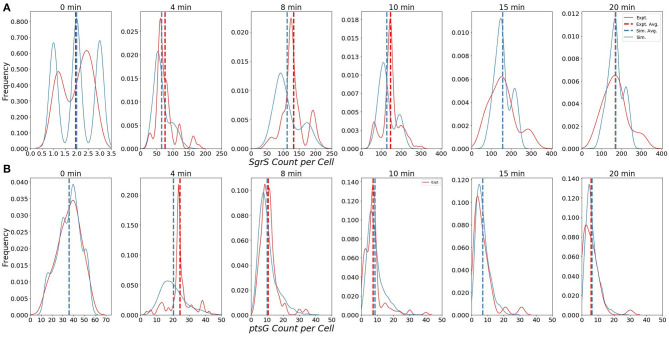
Distributions of **(A)** Wild-Type SgrS (top) and **(B)** *ptsG* mRNA (bottom) at various time points from 0 to 20 min post-induction. Data from smFISH-STORM experiments (red, 100–200 cells per time point) and stochastic simulations (blue, 2,000 cells per time point) are shown as kernel density estimates. The effect of number of cell replicates is studied further in Supplementary Figure 3. Average copy number at each time point is are displayed with dashed vertical lines.

**Figure 7:**
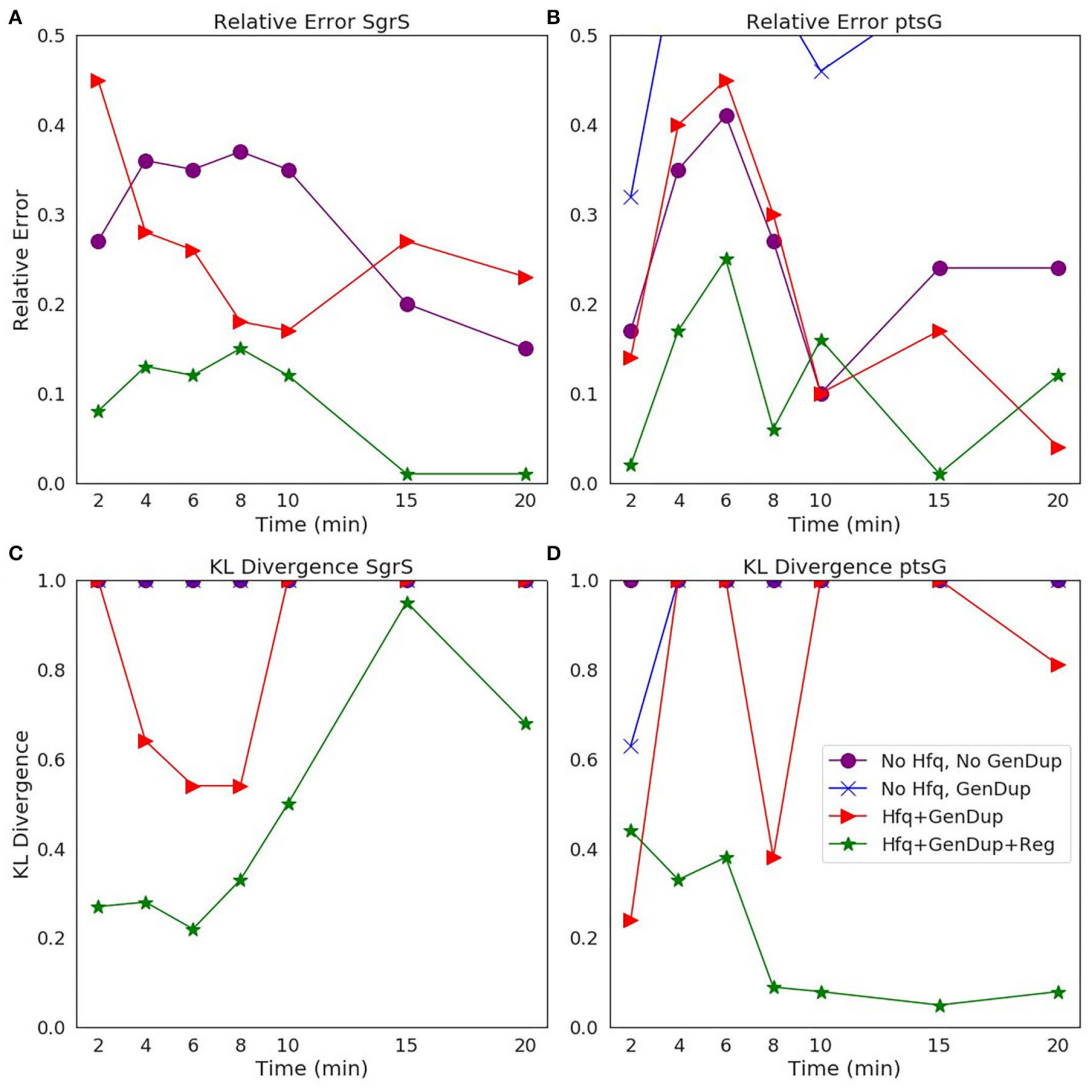
Statistical analysis of the agreement of **(A,C)** *SgrS* sRNA and **(B,D)** *ptsG* mRNA copy number between experiment and theory on both **(A,B)** an average (Relative Error) and **(C,D)** distribution (Kullback–Leibler: KL Divergence) level. KL Divergence values for the model with no Hfq stabilization nor Gene Duplication are not shown as the values obtained are at 1.0, corresponding to significant disagreement in that model variant and experiment. GeneDup refers to a model with Gene Duplication for both SgrS and *ptsG* implemented and Reg refers to a model with transcriptional regulation of SgrS by SgrR in place. The green line (with star markers) indicates the full kinetic model used for this study, which provides the best fit to both average and population level data for both SgrS and *ptsG* mRNA.

The ability of our improved kinetic model to capture population-level statistics of single cell copy number distributions of SgrS and *ptsG* mRNA is demonstrated in Figure 6. Kernel Density Estimates (KDE), which are used to estimate the probability densities of distributions of approximately 100–200 experimentally measured cells and 2,000 simulated cells are displayed, along with dashed vertical lines giving the average RNA copy numbers observed. KDEs were utilized to provide a reasonable comparison to the experimental values despite the fact that there were a relatively low number of cells measured at each time point (approximately 100–200) compared to the number of replicates required for appropriate stochastic simulation (2,000) (Histograms of experimental RNA counts measured before KDE imposition are given in Supplementary Figure 7). The distributions obtained from both experiment and the kinetic model show strong agreement (especially in the case of *ptsG* mRNA), which can be seen quantitatively by the starred line showing the Kullback–Leibler Divergence (KL Divergence) in Figure 7. The KL Divergence (Equation 2), which was minimized to fit to experimental RNA distributions over all time points, is a statistical measure used to characterize the difference between a probability distribution (the KDE of simulated cells) and a reference distribution (the KDE of experimentally measured cells).

The parameters obtained from the fitting process give some insight into the role of stabilization by Hfq in the SgrS-*ptsG* mRNA target search process and the role of transcriptional regulation by SgrR in the regulatory network. The pseudo first order rate of Hfq binding to SgrS (*k*_*bind*_) is 0.063 ± 0.014 *s*^−1^, while the degradation rate of SgrS (*k*_*ds*_), obtained from Δ*hfq* strain experiments (described in section 2.2), is 0.022 ± 0.002 *s*^−1^. The available Hfq pool size of 250 ± 167 predicted by fitting to the kinetic model seems reasonable in that average proteomics values have been found to be on the order 1,500 (Taniguchi et al., 2010; Santiago-Frangos and Woodson, 2018) and unique sRNAs have been shown to be bound to 10 to 1,000 copies of Hfq in *E. coli* (Melamed et al., 2020) (Further discussion of range of Hfq copy number is given in Supplementary Section 1). Additionally, the aforementioned SgrS-Hfq binding rate *k*_*bind*_ corresponds well to experimentally measured *in vitro* values for sRNA-Hfq binding for sRNA of its approximate size (Fender et al., 2010; Hopkins et al., 2011; Santiago-Frangos and Woodson, 2018). If the pseudo first order rate for *k*_*bind*_ reported in Table 1 is converted to a bulk second order rate by incorporating the Hfq concentration at the predicted available pool size of 250, we obtain a binding rate of 1.5 × 10^5^ *M*^−1^ *s*^−1^. This value (on the order of 1–3 10^5^ *M*^−1^ *s*^−1^ within the uncertainty reported in Table 1) agrees better with the reported value of approximately (Santiago-Frangos and Woodson, 2018) 10^6^ *M*^−1^ *s*^−1^ for long RNAs binding to Hfq (Lease and Woodson, 2004; Fender et al., 2010) than 10^8^ *M*^−1^ *s*^−1^ reported for short, unstructured RNAs binding to Hfq (Hopkins et al., 2011). Since SgrS is a relatively long sRNA (sRNA have typically been found to be between 37 and 300 nt Wang et al., 2015a with a length of 227 nucleotides, the slow sRNA-Hfq binding rate obtained by fitting seems appropriate. This type of slow sRNA association process has been suggested to be characterized by RNA restructuring (by which Hfq remodels sRNA regions in order to make its secondary structure more accessible for target mRNA base pairing) (Antal et al., 2004; Soper and Woodson, 2008; Soper et al., 2011; Bordeau and Felden, 2014), which has been proposed to occur for SgrS (Maki et al., 2010). *k*_*bind*_ is also much greater than the Hfq-SgrS unbinding rate (*k*_*unbind*_) of 0.0018 ± 0.0004 *s*^−1^ which was obtained from fitting to the degradation rate of SgrS in a cell where Hfq was expressed (distinct from the Δ*hfq* rate) by assuming that Hfq-SgrS unbinding is the rate-limiting step in the degradation of free SgrS represented in Figure 4 (**Rxn 2.2**). These results seem reasonable in that SgrS should associate with Hfq at a rate comparable to its degradation as well as that SgrS-Hfq binding should happen at a significantly higher rate than their dissociation for sRNA chaperone stabilization by Hfq to be effective.

The kinetic values for transcriptional regulation by the activator SgrR also seem reasonable with a *k*_*on,Ds*_ of 3.0 × 10^−2^ *s*^−1^ and a *k*_*off, Ds*_ of 9.5 × 10^−3^ *s*^−1^. The gene switching parameters correspond to *sgrS* activation via SgrR binding occurring approximately 30 s after initiation of induction, with all *sgrS* genes assumed to start in the “OFF” state (the effect of starting genes in the “OFF” vs. the “ON” state is analyzed in Supplementary Figure 2). This seems reasonable since SgrS sRNA moves from a basal level of a few copies to greater than 40 copies on average in 2 min time (Figure 5). The fact that *k*_*on,Ds*_ is 3 times greater than *k*_*off,Ds*_ means that activation happens more frequently than deactivation from unbinding of SgrR. This relative behavior is somewhat expected as sugar shock has been induced and SgrR is believed to be transformed to its active conformation as a transcription factor for *sgrS* by binding to a small molecule at its C-terminus (Vanderpool and Gottesman, 2004, 2007). While the available evidence suggests that the activity of SgrR due to solute binding rather than *sgrR* expression affects activation of *sgrS*, it has been demonstrated that SgrR is negatively autoregulated (Vanderpool and Gottesman, 2007) which may lead to a ceiling on the level of *sgrS* activation that can occur even after glucose-phosphate stress is fully induced. Thus, we incorporate constant rates of *k*_*on, Ds*_ and *k*_*off, Ds*_ for *sgrS* activation in our model, instead of a time variant rate constant for either parameter.

### 3.1. Comparison of Goodness of Fit Based on Model Complexity

To illustrate the improvement of the kinetic model to describe cellular populations, we compare simulation results sequentially as each level of complexity (i.e., transcriptional regulation by SgrR, gene replication, and stabilization by the chaperone protein Hfq) is added to the original reduced model presented in Fei et al. (2015). Figure 7 demonstrates the improvement in descriptiveness at both an average and population level with progression to a more fine-grained kinetic model. The relative error (Equation 1) of the average copy number of SgrS and *ptsG* mRNA gives the capability of the model to reproduce experiments on an average level, while the Kullback-Leibler Divergence (KL Divergence) (Equation 2) shows the agreement between the experimentally observed and simulation distributions of RNA copy numbers at a series of times from 0 to 20 min post induction.

The Relative Error used to illustrate the agreement between the experimentally measured average RNA copy number and the theoretical value is given by:

(1) $$\begin{array}{l} \eta =\left|\frac{Exp_{avg}-Sim_{avg}}{Exp_{avg}}\right| \end{array}$$

where *Exp*_*avg*_ is the experimentally measured average RNA copy number at a given time point and *Sim*_*avg*_ is the simulated average RNA copy number at the same time point.

The KL Divergence used to compare agreement between experimental and simulated distributions is given by:

(2) $$\begin{array}{l} D_{KL}\left(P\|Q\right)=\underset{i}{\sum }P\left(i\right)\log\frac{P\left(i\right)}{Q\left(i\right)} \end{array}$$

where *P*(*i*) is the continuous probability distribution given by the Gaussian KDE of the experimental copy number distribution of RNA (SgrS or *ptsG* mRNA) and *Q*(*i*) is the analogous simulated RNA copy number distribution.

It is clear that the decrease in the KL Divergence (Figures 7C,D), describing the ability of the kinetic model to accurately describe cell-to-cell variation, is most substantial in the final model presented in this work (star markers). Accounting for transcriptional regulation by SgrR, ongoing gene replication, and the stabilizing effect of Hfq allows for a more faithful description of the noise observed in a cellular population in the process of sugar shock response.

### 3.2. Characterizing the Effects of SgrS Point Mutation on Association to Hfq and ptsG *mRNA*

The stochastic model we have presented can also be utilized to characterize the effects of *sgrS* point mutations on the regulatory network as a whole. The polyU tail region of *sgrS* comprising the final 8 residues of the 5' end (all of which are uridine in the sRNA) has previously been shown to be an important site for Hfq recruitment (Otaka et al., 2011). When the polyU tail is truncated or similarly disrupted, there is a noticeable decrease in SgrS regulatory efficiency. With this in mind, we used the previously defined kinetic model (See Figure 4) to characterize the effect of a point mutation resulting in a U to G change in SgrS at position 224 (in the polyU tail region, hereafter referred to as U224G) of the sRNA on regulatory kinetics. This point mutation is well downstream of the seed region (nucleotides 168–187) where SgrS-*ptsG* mRNA base pairing occurs (Maki et al., 2010; Bobrovskyy and Vanderpool, 2014) so it should not directly interfere with sRNA-mRNA interactions. It is also important to consider the possible structural effects arising from polyU tail mutation. Through *in silico* folding with the RNA structure prediction tool mFold (Zuker, 2003), we confirmed that the stability of the U224G with a ΔG of −17.60 *kcal*/*mol* is unchanged from the predicted wild-type value of −17.60 *kcal*/*mol*, and also indicated that sRNA structure is conserved Supplementary Figure 5) and the measured wild-type ΔHfq degradation rate (see section 2.2) is appropriate for use in fitting the U224G mutant data (as a rate for Figure 1, **rxn 2.2**).

**Figure 8:**
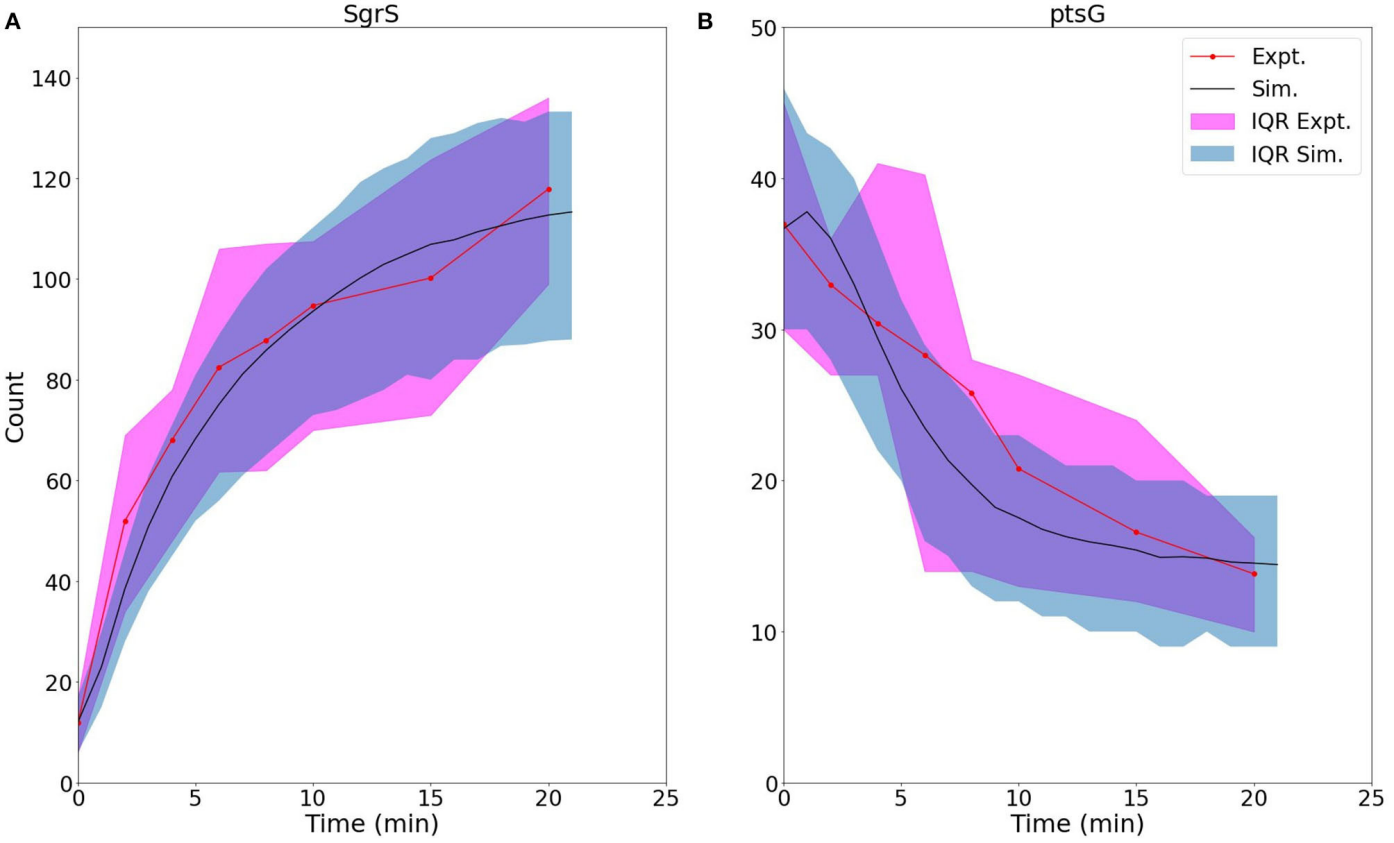
For U224G mutant cells, average time trace and interquartile range (IQR) of **(A)** labeled SgrS and **(B)** *ptsG* mRNA from both 83–110 cells from smFISH experiments (red, circled line) and 2,000 replicates from kinetic model simulations (blue, solid line). The kinetic model shows strong agreement, especially at long times (10–20 min) after induction and captures overall response behavior. An available pool of 250 Hfq and the kinetic parameters given in Table 1 were utilized, other than changes to SgrS-Hfq binding and unbinding rates and *ptsG* mRNA annealing and dissociation rates given in Table 2.

We then fit to the experimentally measured SgrS and *ptsG* mRNA distributions using the previously determined kinetic model (Given in Figure 1 and Table 1). A robust fit describing both average behavior as well as population level variation (Figure 8, Supplementary Figure 4) was achieved primarily by modulating the rates of SgrS to Hfq binding and unbinding (*k*_*bind*_ and *k*_*unbind*_) and the *ptsG* mRNA annealing rates *k*_*on*_ and *k*_*off*_ (which were also free parameters in this treatment) to a much lesser extent, which further demonstrates the role of the polyU tail in Hfq chaperone recruitment. The changes in the kinetic parameters of the model used to fit mutant U224G relative to the wild-type cells (WT) illustrate that the effects of this mutation on SgrS-Hfq association are much larger, relative to the subsequent annealing of SgrS to its target *ptsG* mRNA (Table 2) (Further discussion of mutant U224 structure is given in Supplementary Section 4).

**Table 2:** The list of kinetic parameters for SgrS-Hfq association (*k*_*bind*_ and *k*_*unbind*_) and annealing with *ptsG* mRNA (*k*_*on*_ and *k*_*off*_) for wild-type (WT) cells as well as SgrS mutant U224G (Reactions in Figure 4).

**Parameter**	**Mutant**	**Value**	**% Difference from WT**
*k*_*bind*_	U224G	0.033 ± 0.010 *s*^−1^	−48%
	WT	0.063 ± 0.014 *s*^−1^	
*k*_*unbind*_	U224G	0.003 ± 0.002 *s*^−1^	+66%
	WT	0.0018 ± 0.0004 *s*^−1^	
*k*_*on*_	U224G	(2.1 ± 1.0) ×10^−4^ *molec*^−1^ *s*^−1^	−32%
	WT	(3.1± 0.2) ×10^−4^ *molec*^−1^ *s*^−1^	
*k*_*off*_	U224G	0.27 ± 0.11 *s*^−1^	+22%
	WT	0.22 ± 0.02 *s*^−1^	

The substantial differences between WT and U224G for the values of k_bind_ and k_unbind_ demonstrate the disruption of Hfq binding that accompanies the mutation in the polyU tail, which has been observed previously (Otaka et al., 2011). The smaller relative changes in the ptsG mRNA annealing rates may be due to disruption of RNA restructuring (Antal et al., 2004; Soper and Woodson, 2008; Soper et al., 2011; Bordeau and Felden, 2014) of SgrS by Hfq that hampers association to the mRNA target. Calculation and analysis of parameter uncertainty values by Markov Chain Monte Carlo analysis is discussed in Supplementary Section 6.

The 48% decrease in the SgrS-Hfq binding rate *k*_*bind*_ and 66% increase in the unbinding rate of the sRNA and chaperone complex *k*_*unbind*_ highlight the effects of polyU tail disruption, and support previous conclusions that this is an important site for Hfq stabilization of SgrS (Otaka et al., 2011), and the regulatory efficiency of the network as a whole. The smaller relative changes in the Hfq-SgrS-*ptsG* mRNA annealing rates *k*_*on*_ and *k*_*off*_ by 32% and 22% respectively may be due to altered interactions with Hfq that impair Hfq–dependent annealing of SgrS and *ptsG* mRNA (Supplementary Section 4). In light of the previously discussed slow SgrS–Hfq association process, it is reasonable that RNA restructuring of Hfq may be disrupted by mutation U224G, thus leading to slower and weaker annealing to *ptsG* mRNA. One possible explanation for the disturbance of regulation in mutant U224G is the disruption of orderly transcription termination (the polyU tail is at the 3' end of *sgrS*). Such readthrough transcription has previously been ascribed to decrease the efficiency of SgrS binding to Hfq (Morita et al., 2015, 2017). Even considering values near the ceiling of the uncertainties reported in Table 2 it seems clear that both *k*_*bind*_ and *k*_*on*_ decrease and that both *k*_*unbind*_ and *k*_*off*_ increase due to the disruption of the polyU tail at U224G, highlighting the importance of Hfq in both stabilizing SgrS and in promoting the association of SgrS to *ptsG* mRNA.

## 4. Discussion

The construction of a stochastic kinetic model including gene replication, transcriptional regulation, and the role of the Hfq chaperone protein demonstrates the utility of combining single cell experiments with stochastic modeling. The SgrS Regulatory Network is a noisy system characterized by small numbers of sRNA and mRNA, as well as gene copy numbers that vary from cell-to-cell. This leads to the population level heterogeneity that can then be used to parameterize a kinetic model for analysis of the role of specific molecular actors, such as the chaperone Hfq, and the effects of point mutation on sRNA silencing of mRNA.

The average number of Hfq hexamers present in an *E. coli* cell has been reported to be on the order of 1,400–10,000 (2–15 μM) (Taniguchi et al., 2010; Mancuso et al., 2012; Wiśniewski and Rakus, 2014; Wang et al., 2015b; Santiago-Frangos and Woodson, 2018). It is worth noting that an extensive microfluidic-based, single-cell proteomics study that analyzed over 4,000 individual *E. coli* cells grown in similar media conditions as our study (Taniguchi et al., 2010) found a mean Hfq level of 1500. Additional immunoprecipitation and sequencing studies (by RIL-Seq) have shown the number of various individual mRNAs and sRNAs being bound to Hfq to range from 10 to 1,000 in *E. coli* (Melamed et al., 2020). Thus, our prediction (from fitting) that a pool of approximately 250 ± 167 Hfq (approximately 0.5 μM) are available to bind with SgrS sRNA at any time in the simulation of sugar shock regulation seems reasonable.

In addition, our approach allowed us to characterize the rate of Hfq-SgrS association compared to values reported for Hfq stabilization of other regulatory sRNAs. If the pseudo first order Hfq binding rate *k*_*bind*_ reported in Table 1 is converted to a bulk second order rate we obtain a binding rate of 1.5 × 10^5^ *M*^−1^ *s*^−1^ which agrees reasonably well with the reported value (Santiago-Frangos and Woodson, 2018) of approximately 10^6^ *M*^−1^ *s*^−1^ for long RNAs binding to Hfq (Lease and Woodson, 2004; Fender et al., 2010) (compared to the value of to 10^8^ *M*^−1^ *s*^−1^ for short, unstructured RNAs binding to Hfq Hopkins et al., 2011). SgrS is a relatively long sRNA with a length of 227 nucleotides (sRNAs have been observed with 37-300 nt Wang et al., 2015a), therefore the slow sRNA-Hfq binding process that we describe does seem likely. We suggest that this could be due to RNA restructuring of SgrS (Antal et al., 2004; Soper and Woodson, 2008; Maki et al., 2010; Soper et al., 2011; Bordeau and Felden, 2014) by Hfq in order to promote binding with *ptsG* mRNA. It is thought that cellular sRNA and mRNA are present in large excess over Hfq (Wagner, 2013), so nearly all cellular Hfq hexamers are thought to be bound to RNA. Since cellular mRNA in *E. coli* are found to be on the order of approximately 2,000–8,000 copies (Bartholomäus et al., 2016) (much greater than the highest measured SgrS sRNA value of 200) the available Hfq pool size that we present is representative of the relative competitiveness (and time-dependent cycling) of SgrS for Hfq relative to the other particles that interact with the chaperone.

The study of mutant U224G shows the importance of Hfq stabilization in the SgrS regulatory network as a whole and seems to corroborate previous findings (Otaka et al., 2011) that highlight the importance of the polyU tail for Hfq association with SgrS. The substantial decrease of the Hfq-SgrS binding rate and increase in the related unbinding rate relative to the *ptsG* mRNA annealing rates further down the network obtained from fitting confirms this point (Table 2). The changes in the SgrS-*ptsG* mRNA annealing rates *k*_*on*_ and *k*_*off*_ seem to support conclusions from the wild-type cells that Hfq-SgrS binding may result in some restructuring of the sRNA that makes this a slow process. This may explain the lower efficiency in *ptsG* mRNA association observed in mutant U224G, since Hfq cannot bind SgrS as effectively due to mutation at the polyU tail. Therefore, the predicted restructuring of SgrS by Hfq necessary to facilitate *ptsG* mRNA association is also hampered, resulting in slower and less stable mRNA binding (a decrease in *k*_*on*_ and an increase in *k*_*off*_).

While this work is useful in describing the role of Hfq in the SgrS regulatory network and in capturing the stochastic nature of regulation over a population of replicating cells, it does not consider certain cellular processes that may affect network dynamics. First, the various other SgrS mRNA targets that may be present in a living *E. coli* cell under certain growth conditions may affect the SgrS pool available to regulate *ptsG* mRNA. In addition, other factors such as sRNA recycling (i.e., SgrS not being co-degraded with its target mRNA) (Soper et al., 2011; Santiago-Frangos and Woodson, 2018), which have been proposed for some sRNA and are now under study for SgrS, were not included, but can be incorporated into the model. Also, the process of spatial target search (via RNA and protein diffusion) of SgrS-Hfq for *ptsG* mRNA and RNase E (which may be localized in ribonucleoprotein bodies Al-Husini et al., 2018 or near the membrane Moffitt et al., 2016) for the entire protein-RNA complex as it seeks to degrade the RNA is not explicitly considered in our model (as it a well-stirred model). The potential of binding of the SgrS to *ptsG* mRNA as soon as the sRNA binding site on the mRNA is transcribed [i.e., co-transcriptional regulation which has been posited previously by Chen et al. (2019)], may be of interest to add to the model, since the model assumes only post-transcriptional binding of *ptsG* mRNA to the SgrS-Hfq complex. A further experiment that would be useful in the study of these processes would be an RIL-Seq experiment (Melamed et al., 2020) that quantifies the interactions of Hfq with other RNA (such as *yigL* or *manX*) relative to its interactions with SgrS, to better understand the pool of Hfq available for the SgrS stress response network.

In conclusion, by incorporating gene replication, stabilization by the chaperone protein Hfq, and transcriptional gene regulation of *sgrS* we have developed a kinetic model capable of describing the cellular heterogeneity observed in the *E. coli* sugar shock response network. Stochastic simulation of the kinetic model allows us to take full advantage of the single-molecule fluorescence data that illustrates cell-to-cell variability in a collection of hundreds of cells. While the post-transcriptional regulation and silencing of *ptsG* mRNA by the sRNA is the critical feature, accounting for gene replication, transcriptional regulation, and stabilization gives a more robust picture of the regulatory network as a whole. In addition, complexifying the model highlights the importance of stabilization by Hfq and chaperone proteins in general in RNA silencing networks and allowed for a prediction of the rate of association of SgrS and Hfq (as a slow process, characterized by restructuring), the effective available Hfq pool size for the SgrS regulon under sugar stress conditions, as well as an analysis of an SgrS point mutation in one of the presumed Hfq binding modules (the polyU tail). The model presented in this work establishes a framework for models analyzing other sRNA mediated gene regulatory networks, and can be extended to spatially-resolved models describing SgrS target search kinetics.

## Data Availability Statement

The original contributions presented in the study are included in the article/Supplementary Material, further inquiries can be directed to the corresponding author/s. The Jupyter notebook containing the model used in this study as well as plotting and analysis of sample stochastic simulations can be found at: http://faculty.scs.illinois.edu/schulten/research/sgrs-2020/. Experimental smFISH-STORM data is also available at the preceding web address and is shown in Supplementary Data Sheet 1.

## Author Contributions

DB, TB, and ZL-S: writing–original draft and writing–reviewing and editing. AP: performed single-molecule experiments. MA: cultivated cell strains and performed sRNA lifetime experiments. DB: design and simulation of stochastic model. TB: writing of Jupyter notebook. AP, MA, CV, and TH: reviewing. DB, TB, AP, ZL-S, CV, and TH: conceived research plan. All authors contributed to the article and approved the submitted version.

## Conflict of Interest

The authors declare that the research was conducted in the absence of any commercial or financial relationships that could be construed as a potential conflict of interest.
