# Supplementary material for: Stochastic Analysis Demonstrates the Dual Role of Hfq in Chaperoning E. coli Sugar Shock Response
Source: Front Mol Biosci. 2020 Dec 23;7:593826. doi: 10.3389/fmolb.2020.593826 (PMC7786190; doi:10.3389/fmolb.2020.593826)
Supplement: Supplementary file 1 [file Data_Sheet_1.PDF]

Supplementary Material

1 EFFECTS OF VARYING AVAILABLE HFQ POOL SIZE

The available pool of Hfq utilized in the model represents the fraction of cellular Hfq hexamers bound to SgrS as opposed to other targets and thus the relative binding strength of SgrS compared to other RNAs stabilized by the chaperone. Previous work (Melamed et al., 2020) has shown that the typical number of Hfq bound to a given sRNA varies widely across sRNA species. If an even smaller pool of cellular Hfq is assumed to be available for SgrS binding under sugar shock conditions the average behavior of SgrS experimentally observed can be more exactly captured (Figure S1). However, this comes at a loss of the population level noise observed in the measured RNA distributions because fewer SgrS can be stabilized and so it decays on a much faster timescale, resulting in a loss of cell-to-cell variation. When additional Hfq is added to the available pool such as the 800 available in the simulations shown in Figure S1 the opposite behavior can be seen. SgrS exhibits greater population level heterogeneity, but with a less robust fit to the average behavior that is experimentally observed. We propose that the presence of more Hfq leads to more sRNA noise because SgrS is less likely to be present in its free form and decays more slowly when it is associated with *ptsG* mRNA and Hfq (k_{on} is small relative to k_{ds}) than it would when it is not stabilized by Hfq (**Figure 4**, **rxn 2.2** versus **rxn 4** followed by **rxn 5**).

2 EFFECTS OF INITIAL GENE STATE

Of interest from a more technical standpoint, is the state of the *sgrS* genes at time = 0 minutes in the simulation. While, in principle these genes should be in the "OFF" state and unable to be transcribed since induction has yet to begin it is interesting to understand the effects of initial gene state on population level noise. Consider the following example, when all SgrS genes begin in the "ON" state. While the average behavior at times from 4 to 10 minutes is poorly captured, the RNA distributions are well-described at 15 and 20 minutes post-induction (Figure S2). This example assumes an immediate switch from the "OFF"

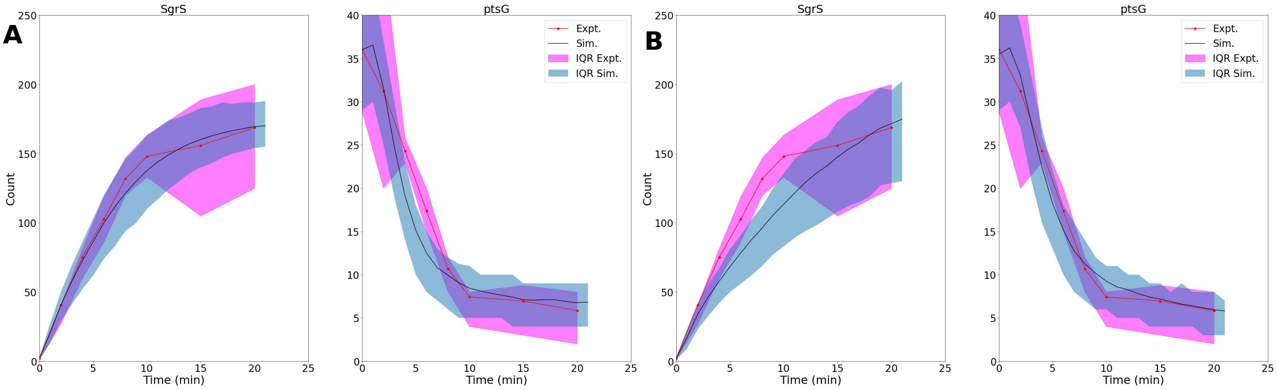

Figure S1. (**A**): Trace and interquartile range (IQR) of SgrS sRNA and *ptsG* mRNA mRNA where simulations include a smaller pool of 200 Hfq available (versus 250 in main text simulations). While averages can be more tightly fit, the population level variation observed for SgrS is minimized even further from what is observed experimentally, including at long times post-induction. (**B**): A similar plot of Trace and IQR with Hfq available pool size equal to 800. Here the population level variation is larger (especially at long times post induction), but the initial average traces are less well captured.

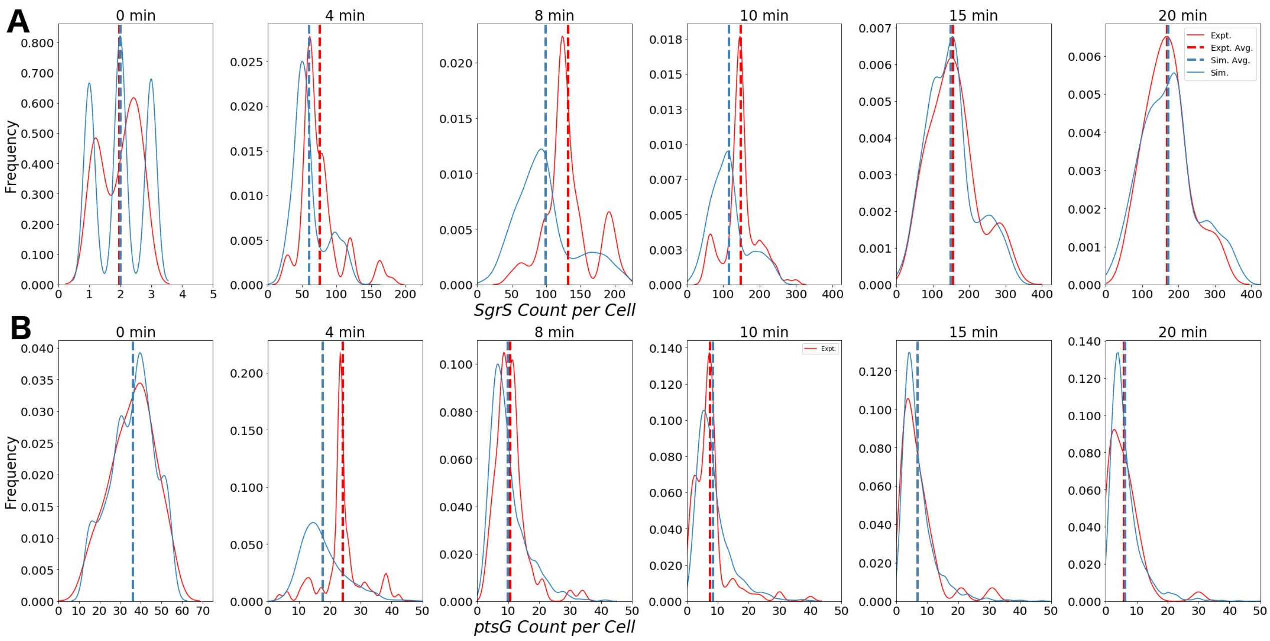

Figure S2. Distributions of **(A)** Wild-Type SgrS and **(B)** *ptsG* mRNA (bottom) at various time points from 0 to 20 minutes post-induction for cells with all genes starting in the "ON" state. Data from smFISH–STORM experiments (red, 100-200 cells per time point) and stochastic simulations (blue, 2000 cells per time point) are shown as kernel density estimates. Average copy number at each time point is displayed with dashed vertical lines.

to the "ON" state of the *sgrS* genes due to induction. While unrealistic when taken at face value, it is reasonable to assume the induction occurs on the order of seconds, since the amount of SgrS increases by a factor of 10 from its basal value by 2 minutes post-induction (**Figure 5**) and since binding of the SgrR activator for *sgrS* is mediated by binding to a small molecule (*i.e* sugar), which presumably takes some interval of time. The smaller $k_{on,Ds}$ and $k_{off,Ds}$ values ($2.0 \times 10^{-3}\ s^{-1}$ and $6.5 \times 10^{-4}\ s^{-1}$ versus $3.0 \times 10^{-2}\ s^{-1}$ and $9.5 \times 10^{-3}\ s^{-1}$, respectively) used in **Figure 4** Rxn 2.0 relative to those given in Table 1 then lead to a wider range of population distributions at late times due to longer dwell times (*i.e.* up to 5 minutes) for the *sgrS* gene in the "OFF" state compared to the a typical dwell time of less than 1 minute in the "OFF" state when the more appropriate regulatory values ($3.0 \times 10^{-2}\ s^{-1}$ and $9.5 \times 10^{-3}\ s^{-1}$, based on the rapid increase in SgrS copy number from 0 to 2 minutes) are used for $k_{on,Ds}$ and $k_{off,Ds}$ respectively.

3 EFFECTS OF INCREASED CELL REPLICATE NUMBER

The number of *E. coli* cells that are simulated or have their RNA distributions experimentally measured is of great importance when considering a process characterized by stochasticity. A certain number of cells must be observed to accurately capture both the average behavior and cell-to-cell variability that emanates from a kinetic regulatory system (Taniguchi et al., 2010; Elowitz et al., 2002; Raser, 2005).

Figure S3 shows the effect of number of cells measured on the average and standard deviation of the SgrS simulated at 20 minutes post-induction. The bootstrapping technique presented allows for the selection of an individual *E. coli* cellular replicate, with replacement, up to N cells. The vertical dashed line in each figure shows the expected average and standard deviation values produced from bootstrapping with N=85, the number of cells experimentally measured at time 20 minutes post-induction. This highlights

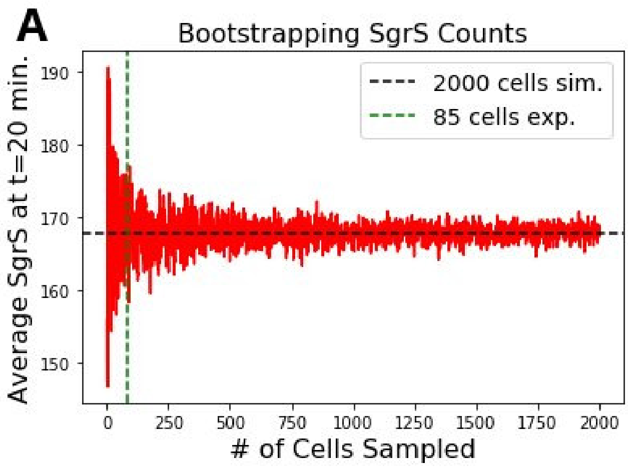

Figure S3. Bootstrapping of SgrS sRNA simulated at 20 minutes post sugar shock induction showing the variation in the **(A)** population mean and **(B)** population standard deviation with number of simulated cells sampled. The x axis gives the number of samples taken (N) with replacement out of a total 2000 independent simulation trajectories in the bootstrapping procedure. The vertical dashed line at N=85 shows the number of cells experimentally imaged at this time point. It takes several hundred to 1000 simulated cells before the SgrS mean and population level variation noise begin to relax to the calculated values.

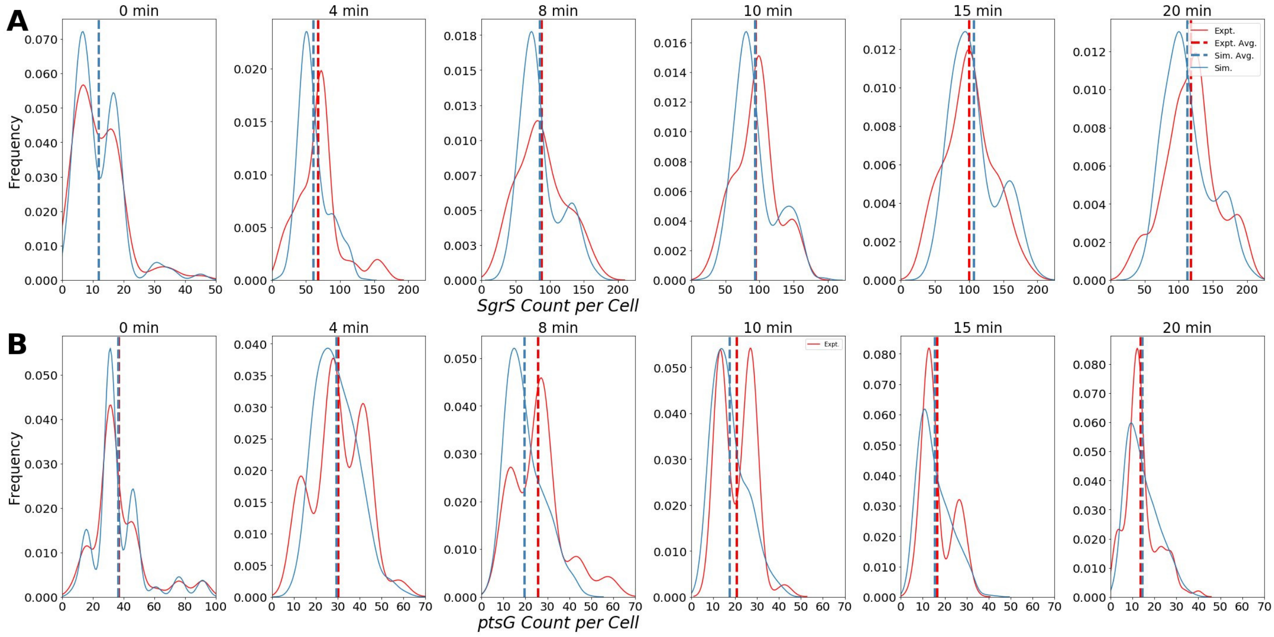

Figure S4. Distributions of the SgrS polyU tail mutant U224G for **(A)** SgrS and **(B)** *ptsG* mRNA at various time points from 0 to 20 minutes post-induction. Data from smFISH–STORM experiments (red, 100-200 cells per time point) and stochastic simulations (blue, 2000 cells per time point) are shown as kernel density estimates. Average copy number at each time point is are displayed with dashed vertical lines.

the possible error in both mean copy number (5-10 copies) or population level variation (5-10 copies) that could be accrued due to insufficient sampling.

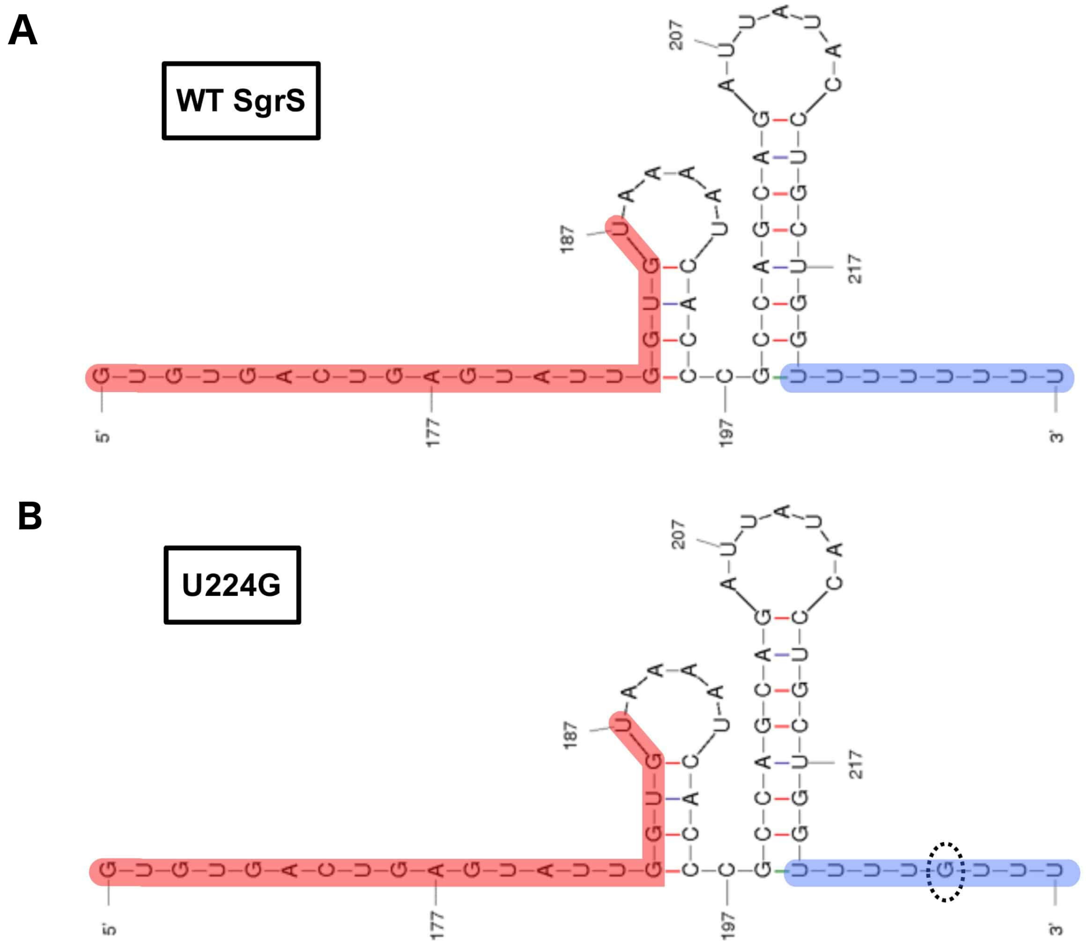

Figure S5. Flattened predicted sRNA structures for **(A)** wild-type (WT) SgrS as well as **(B)** the U224G mutant studied in this work obtained via mFold *in silico* folding. **Red**: the SgrS-*ptsG* mRNA baseparing region, **Blue**: the polyU tail, with the mutated residue circled in the U224G structure. The predicted structures show similar conformation as well as identical free energies ($-17.60\ kcal/mol$), indicating that SgrS secondary structure is likely not significantly destabilized by the U224G point mutation in the polyU tail.

4 EFFECTS OF SGRS POINT MUTATION ON REGULATORY KINETICS

In order to fit to mutant U224G the same parameters as for the wild-type cells were utilized other than the SgrS-*ptsG* mRNA binding and unbinding rates k_{bind} and k_{unbind} and the *ptsG* mRNA association rates k_{on} and k_{off}. The same gene state (high versus low gene copy number) percentages for *sgrS* and *ptsG* as for the wild-type cells were arrived at by fitting as well as the same "available" Hfq pool size of 250 hexamers. The distributions (as kernel density estimates) shown in Figure S4 for both SgrS and *ptsG* mRNA were obtained via the same fitting process described in the main text.

In order to focus on a point mutation that primarily showed a disruption in SgrS-Hfq association we sought a mutant in which SgrS secondary structure would not be significantly disrupted, leading to a higher free degradation rate of SgrS. In this way, we can isolate the effects of the point mutation on SgrS association to both the chaperone Hfq and its target *ptsG* mRNA individually. Via *in silico* folding using the RNA structure prediction tool mFold (Zuker, 2003), we confirmed that the stability of the U224G with a ΔG of $-17.60\ kcal/mol$ is unchanged from the predicted wild-type value of $-17.60\ kcal/mol$. The predicted

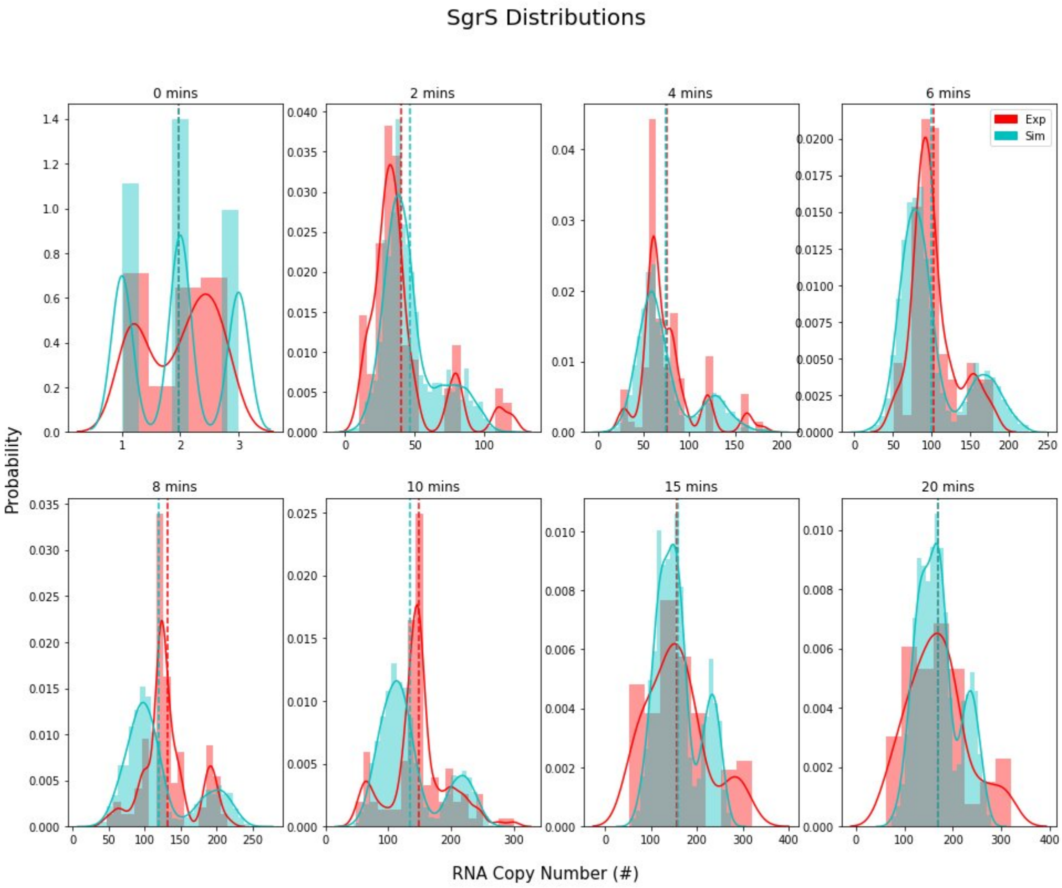

Figure S6. SgrS histograms, with experimental data in **red** and simulation data in **blue**. Scott's normal reference rule was utilized to determine histogram bin widths.

U224G mutant structure also shows similar two stem loop structure (with loops of identical size) to that of the wild-type (Figure S5).Thus, an assumption that the measured wild-type ΔHfq degradation rate (see Main-text Section "Materials and Methods") is appropriate for use as an SgrS-Hfq disassociation rate in fitting the U224G mutant data is reasonable.

5 HISTOGRAMS OF EXPERIMENTALLY DETERMINED RNA COUNTS

The experimental data and simulated data shown in histogram form, prior to conversion to Kernel Density Estimates (KDEs) used in the main text and for fitting and analysis. Scott's normal reference rule (Equation S1) was used to determine the bin width for the histograms of SgrS and *ptsG* mRNA at each time point. It is clear that the imposition of a kernel density on the experimental data, which was useful in constructing a fitting optimization scheme, has not fundamentally altered the character of the experimentally observed population level variation in SgrS or *ptsG* mRNA counts.

$$h = \frac{3.49\sigma}{n^{\frac{1}{3}}} \tag{S1}$$

where h is the bin-width, σ is the sample standard deviation, and n is the number of samples (cells measured).

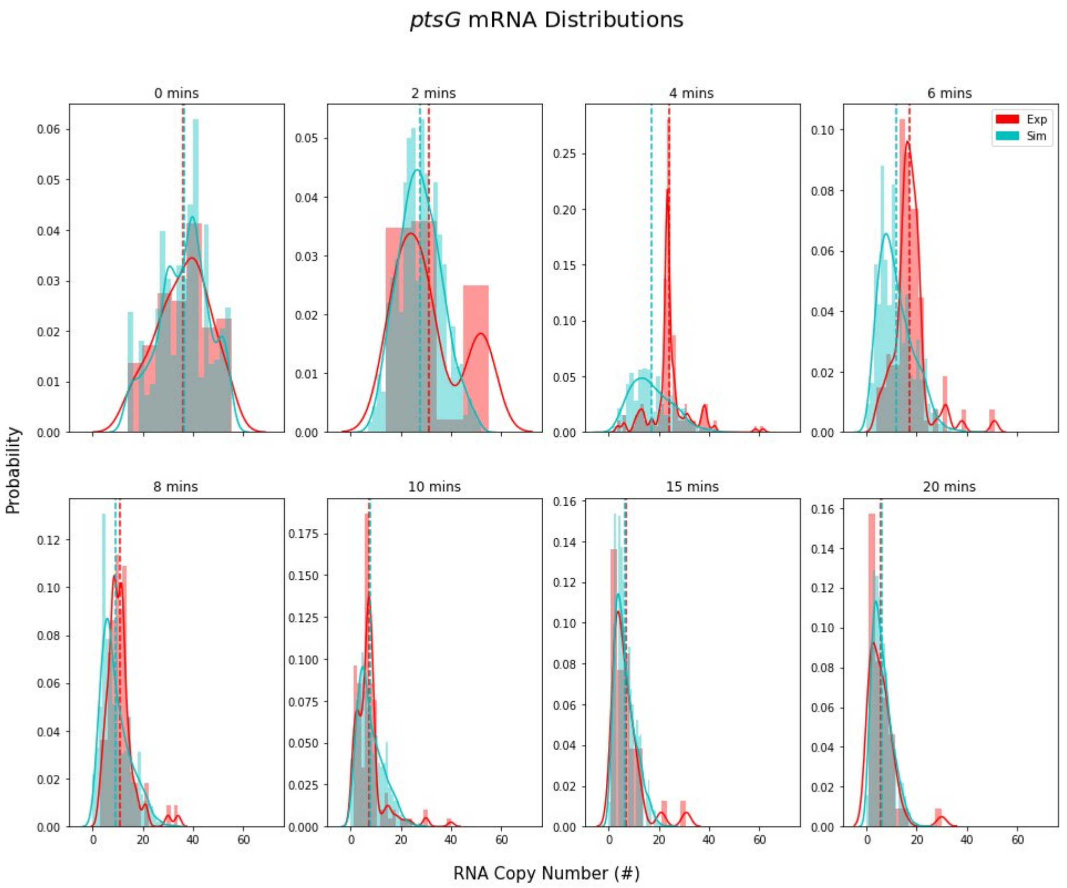

Figure S7. *ptsG* mRNA histograms, with experimental data in **red** and simulation data in **blue**, with associated Gaussian Kernel Density Estimate (KDE) and average values as vertical dashed lines. Scott's normal reference rule was utilized to determine histogram bin widths.

6 ANALYSIS OF UNCERTAINTY IN FITTING MODEL KINETIC PARAMETERS

The sequential process of building the stochastic kinetic model from the existing ODE model presented in Fei et al. (2015) was also useful in quantifying the uncertainty of specific model parameters. After initial fitting of an ODE form of the kinetic model to average SgrS and *ptsG* mRNA counts, we quantified the error in each parameter by using a Markov Chain Monte Carlo (MCMC) approach. Via MCMC, we sampled the parameter space of the more complex stochastic model about the parameters arrived at by fitting stochastically (**Table 1**, **Table 2**) to minimize Kullback-Leibler Divergence between experimental and simulated RNA distributions. MCMC sampling and analysis was performed via the Python emcee package (Foreman-Mackey et al., 2013) implemented in the lmfit Python package (Newville et al., 2014) and with the LSODA ODE solver (Petzold, 1983) being utilized to evaluate the kinetic model for each sampled parameter set. In the MCMC process an ensemble of parameter sets (of the kinetic parameters in the complex stochastic model) evolves to sample a Bayesian posterior distribution, which is the product of a prior distribution and a likelihood function. The logarithm of the likelihood function is given by

$$Ł = ln\left(p(y|x,\theta)\right) = \sum_m \left(-\frac{1}{2}\sum_n \left[\frac{y_{m,n} - f(x_{m,n}|\theta))^2}{\sigma^2_{m,n}}\right]\right) \tag{S2}$$

where m is the molecular species (SgrS, *ptsG* mRNA), n is the time point (8 in our case, t = 0, 2, 4, 6, 8, 10, 15, 20 minutes after stress induction), $y_{m,n}$ is the experimental count value for m at time point n and $f(x_{m,n}|\theta)$ is the simulated value for m at time point n given the parameter set value θ and $\sigma^2_{m,n}$ is the experimental variance for molecular species m at time t_n. The log-posterior distribution is the sum of the log-prior distribution and the log-likelihood function.

We used 100 walkers, (representing 100 parameter sets), each evolved for 5000 iterations, to calculate the error associated with each fit parameter via MCMC. Prior distributions of parameter values were centered uniformly about the parameters values arrived at from stochastic fitting. Values obtained from stochastically fitting to experimental RNA distributions by minimizing KL Divergence were used as initial guesses for each parameter in the MCMC simulation process. The error bounds (the Monte Carlo error from the MCMC fitting process that minimizes the negative log-likelihood given in Equation S2) for each parameter are reported in **Table 1** for WT cells and in **Table 2** for mutant U224G. Errors for the literature values from Fei et al. (2015) and the associated simplified model that was re-fit to the experimentally measured RNA distributions in this work (See main-text Section "Experimental Methods and Materials") are also given in **Table 1** and **Table 2**. Even taking values near the maximum of the uncertainties presented in **Table 1** and **Table 2** the main findings of this study still seem to hold: 1) That SgrS-Hfq binding is a slow process that may be characterized by RNA restructuring, 2) That a pool of 100s of Hfq is available to stabilize SgrS during stress response, 3) That the U224G mutation in the polyU tail, hampers Hfq binding to SgrS and decreases the efficacy of later *ptsG* mRNA association (See main-text Section "Discussion").

In order to further examine these parameter error values, we plotted deterministic solutions to the kinetic model using the maximum and minimum error values added to each parameter. It is clear from this analysis that the qualitative trends that can be observed through the fitting process given in the main text do not

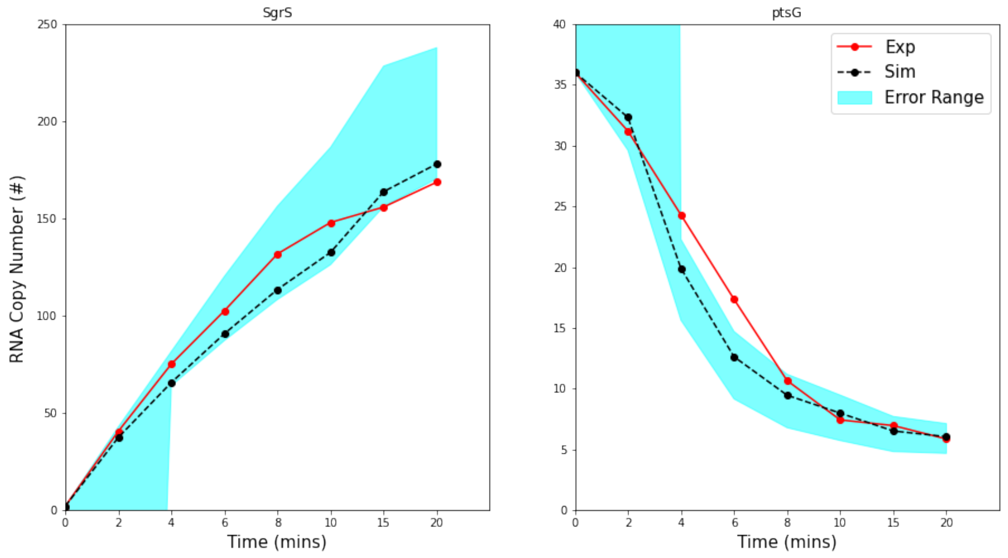

Figure S8. Simulated trace and error range for Wild-Type cells (Extending from the minimal to the maximal values by adding the MCMC errors to the fit parameter value) when varying the SgrS-Hfq binding rate k_{bind} for SgrS and *ptsG* mRNA . Experimental average RNA counts are given in the solid line and simulated average RNA counts are shown via the dashed line with surrounding shading representing the error value range for the parameter.

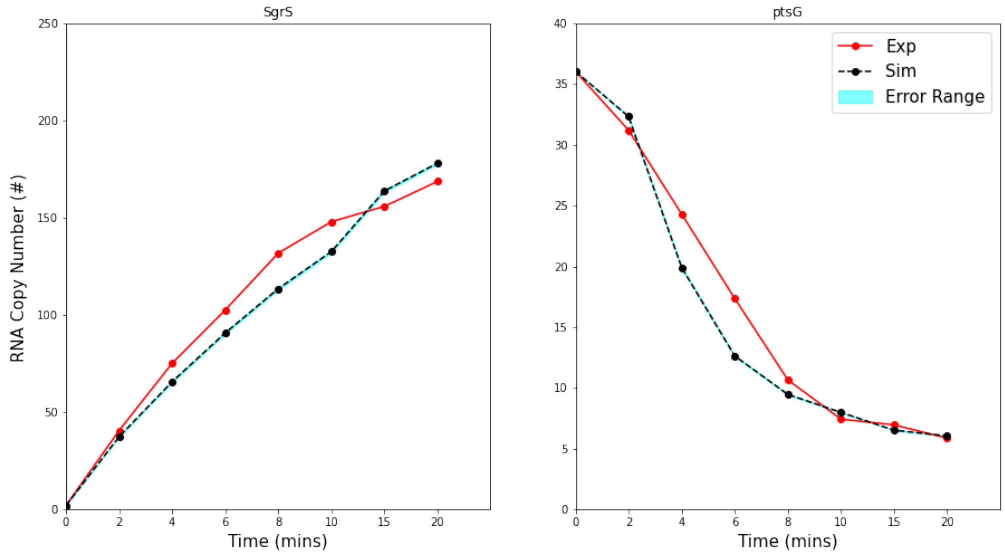

Figure S9. Simulated trace and error range for Wild-Type cells (Extending from the minimal to the maximal values by adding the MCMC errors to the fit parameter value) when varying the *sgrS* "ON" rate for gene transcriptional activation ($k_{on,Ds}$) for SgrS and *ptsG* mRNA. Experimental average RNA counts are given in the solid line and simulated average RNA counts are shown via the dashed line with surrounding shading representing the error value range for the parameter.

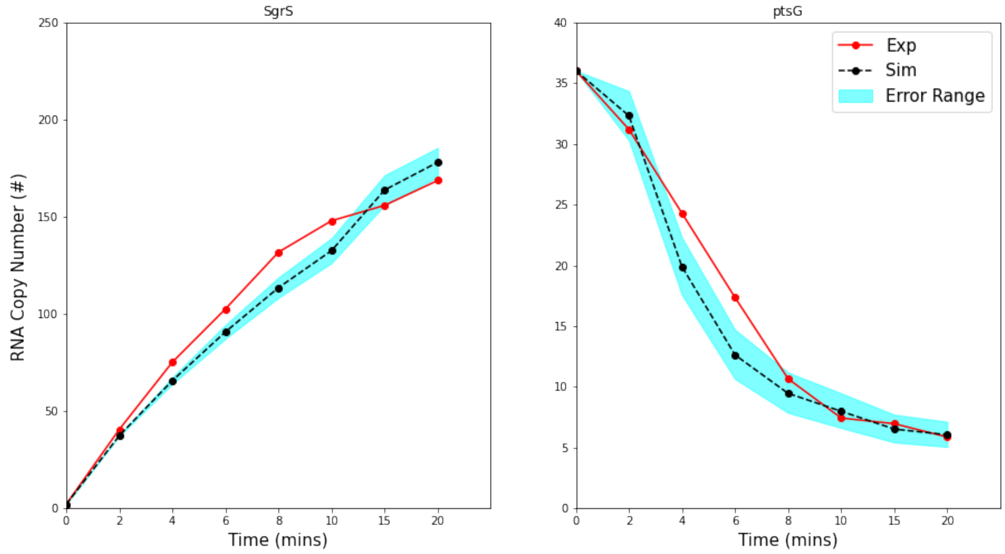

Figure S10. Simulated trace and error range for Wild-Type cells (Extending from the minimal to the maximal values by adding the MCMC errors to the fit parameter value) when varying the high gene state percentage for *ptsG* ($nHighP$) for SgrS and *ptsG* mRNA. Experimental average RNA counts are given in the solid line and simulated average RNA counts are shown via the dashed line with surrounding shading representing the error value range for the parameter.

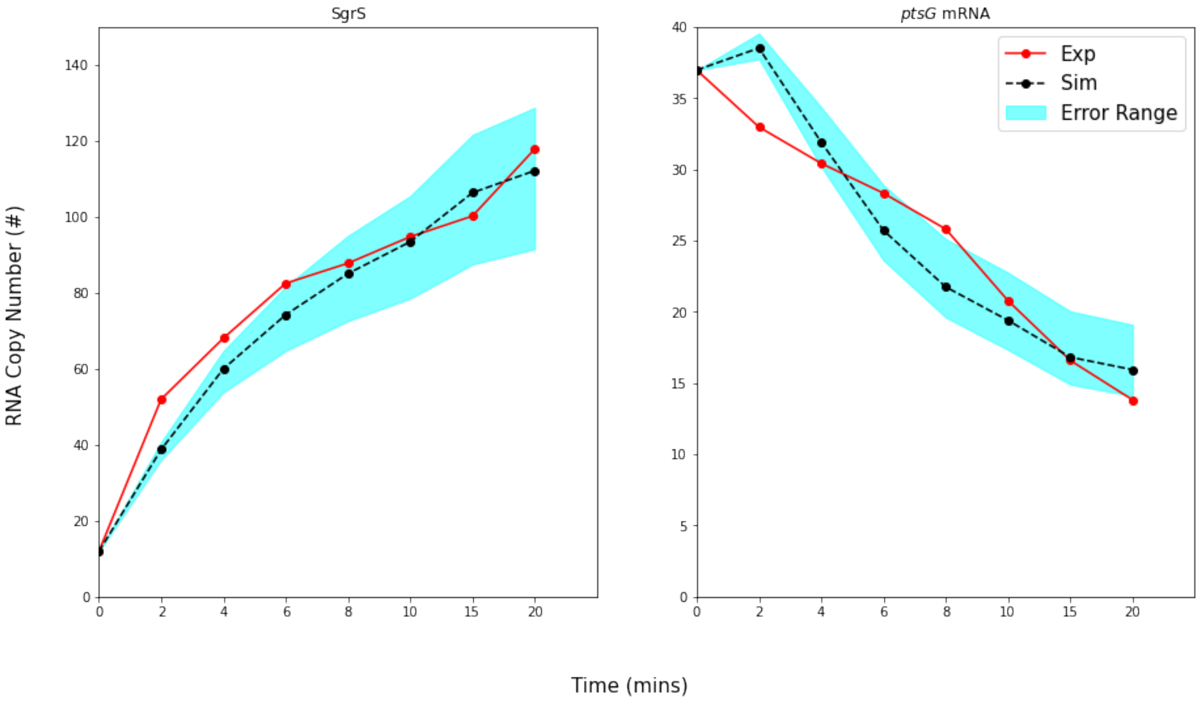

Figure S11. Simulated trace and error range for U224G mutant cells (Extending from the minimal to the maximal values by adding the MCMC errors to the fit parameter value) when varying the SgrS-Hfq binding rate k_{bind} for SgrS and *ptsG* mRNA. Experimental average RNA counts are given in the solid line and simulated average RNA counts are shown via the dashed line with surrounding shading representing the error value range for the parameter.

change. In addition, we can further analyze the relative impact of changes to each individual parameter on the model as a whole. For example, if we take the binding rate of Hfq to SgrS (k_{bind}, see **Figure S8**) for Wild-Type cells, we can see that the maximal and minimal error values give the same qualitative trend to the fit, however they accrue discrepancies from the experimentally measured RNA averages on the order of approximately 50 RNA for SgrS at some time points. As is demonstrated later in this analysis for mutant U224G, the error in k_{bind} has some of the most substantial effects on overall model error since SgrS is significantly stabilized from degradation when it is in its chaperone bound Hfq-SgrS form compared to when it is present as a free sRNA. If we examine a less critical parameter in the model such as the "ON" rate for *sgrS* gene activation (k_{onDs}, see **Figure S9**), even taking the minimal or maximal value does not substantially change the predicted RNA counts in an average sense. It is also interesting to examine the effect of changing the high-state gene percentage ($nHighP$, see **Figure S10**) a critical value in obtaining appropriate stochastic distribution and to observe its somewhat more substantial error from the experimental average that occurs.

For the U224G mutant we also investigated the relative effects of the error bounds on the kinetic model for each of the four parameters affected by this mutation (k_{bind}, k_{on}, k_{off}, k_{unbind}). The SgrS-Hfq binding rate k_{bind} showed the most substantial error range, whose average simulated RNA traces are shown in **Figure S11**. This again highlights the importance of the hampering of SgrS-Hfq association by disruption of the polyU tail of the sRNA.

REFERENCES

Elowitz, M. B., Levine, A. J., Siggia, E. D., and Swain, P. S. (2002). Stochastic gene expression in a single cell. *Science* 297, 1183–1186. doi:10.1126/science.1070919

Fei, J., Singh, D., Zhang, Q., Park, S., Balasubramanian, D., Golding, I., et al. (2015). Determination of in vivo target search kinetics of regulatory noncoding RNA. *Science* 347, 1371–1374. doi:10.1126/science.1258849

Foreman-Mackey, D., Hogg, D. W., Lang, D., and Goodman, J. (2013). emcee: The MCMC hammer. *Publications of the Astronomical Society of the Pacific* 125, 306–312. doi:10.1086/670067

Melamed, S., Adams, P. P., Zhang, A., Zhang, H., and Storz, G. (2020). RNA-RNA interactomes of ProQ and hfq reveal overlapping and competing roles. *Molecular Cell* 77, 411–425.e7. doi:10.1016/j.molcel.2019.10.022

[Dataset] Newville, M., Stensitzki, T., Allen, D. B., and Ingargiola, A. (2014). Lmfit: Non-linear least-square minimization and curve-fitting for python. doi:10.5281/ZENODO.11813

Petzold, L. (1983). Automatic selection of methods for solving stiff and nonstiff systems of ordinary differential equations. *SIAM Journal on Scientific and Statistical Computing* 4, 136–148. doi:10.1137/0904010

Raser, J. M. (2005). Noise in gene expression: Origins, consequences, and control. *Science* 309, 2010–2013. doi:10.1126/science.1105891

Taniguchi, Y., Choi, P. J., Li, G.-W., Chen, H., Babu, M., Hearn, J., et al. (2010). Quantifying e. coli proteome and transcriptome with single-molecule sensitivity in single cells. *Science* 329, 533–538. doi:10.1126/science.1188308

Zuker, M. (2003). Mfold web server for nucleic acid folding and hybridization prediction. *Nucleic Acids Research* 31, 3406–3415. doi:10.1093/nar/gkg595
